# Hypomethylation at non-CpG/CpG sites in the promoter of HIF-1α gene combined with enhanced H3K9Ac modification contribute to maintain higher HIF-1α expression in breast cancer

**DOI:** 10.1038/s41389-019-0135-1

**Published:** 2019-04-02

**Authors:** Chun Li, Wei Xiong, Xiong Liu, Wenjun Xiao, Yuxian Guo, Junyu Tan, Yaochen Li

**Affiliations:** 1 The central laboratory, of Cancer Hospital of Shantou University Medical College, No.7 Raoping Road, Shantou, 515031 China; 2 Department of Pathology, of Cancer Hospital of Shantou University Medical College, No.7 Raoping Road, Shantou, 515031 China

## Abstract

HIF-1α has a broad impact on tumors, including enhanced utilization of glucose, tumor cell stemness, migration, metastasis and so on. In pilot study, we found that the expression of HIF-1α significantly increased in breast cancer cell lines and tissue samples with higher malignant behaviors and decreased in luminal subtype breast cancer cells and tissue samples. We analyzed and found there is one large CpG island in HIF-1α promoter around transcription start site, and the hypermethylation occurred at these CpGs and their surrounding non-CpGs sites. Epigenetic events driving tumorigenesis has been characterized. However, knowledge is lacking on the non-CpGs methylation of HIF-1α promoter in breast cancer cells. We validated that non-CpGs methylation can directly regulate HIF-1α expression by luciferase activity assay. We also found DNMT3a and Mecp2 play vital role in methylation at non-CpGs and CpGs sites. In addition, we noticed that H3K9ac modification could promote the transcription of HIF-1α in MDA-MB-231 cells by binding to the region contained hypomethylated non-CpG and CpG sites. Taken together, the hypomethylation status at non-CpG and CpG loci in HIF-1α promoter and H3K9ac modification together contribute to maintain higher HIF-1αactivity in invasive breast cancer cells when compared with the non-invasive breast cancer cells, which may establish a tissue-specific epigenetic modification pattern and point to the new directions for future understanding breast cancer therapy.

## Introduction

Breast cancer is a global health problem and is one of the leading causes of cancer deaths for women^1,2^. In China, estimates of new breast cancer cases were approximately 278,900 in 2014^3^.

Currently, based on molecular profiling, breast malignant tumors are classified into five major subtypes: basal-like, two luminal-like, normal-like and epidermal growth factor receptor type 2 (HER2) over-expressing cancers^4^.

The triple negative breast cancer (TNBC) is characterized by negative expression of estrogen and progesterone receptors (ER-negative, PR-negative) as well as HER2, and accounts for approximately 16% of all breast cancer diagnoses^5,6^. TNBC is often used as a surrogate for identifying the aggressive basal breast cancer subtype, and although the two patterns share many similarities, they are not biologically synonymous^7^.

Hypoxia has been recognized as a common characteristic in many types of solid tumors, including TNBC. Cancer cells in a hypoxic region begin to adapt to low oxygen tension conditions by activating several survival pathways. Activation of the transcription factor HIF-1 is the most recognized mechanism adopted by hypoxic cells in this harsh microenvironment. HIF-1*α*, as an oxygen sensitive subunit, is induced under hypoxic conditions, and then translocated to the nucleus where it heterodimerizes with HIF-1β subunits to form active HIF-1 protein that binds to specific hypoxic response elements present in target gene promoters, ultimately activating transcription of these genes. As a consequence, HIF-1α has a broad impact on tumor that promotes processes for tumor progression including angiogenesis, autophagy^8,9^, enhanced glucose utilization, tumor cell stemness^10^, EMT, metastasis, and resistance to radiotherapy and chemotherapy^10–14^. In view of this, HIF-1α has become a vital molecular target for breast cancer formation and progression.

The cellular level of HIF-1α is tightly regulated. HIF-1α is maintained at low levels under normoxia conditions by the collaboration between PHD (proline hydroxylase domain) proteins and the VHL-containing E3 ubiquitin ligase complex. In addition to controlling HIF-1α transcriptional activity by regulating HIF-1α stability, other means of regulation include SUMOylaton, acetylation, and phosphorylation^15^.

Herein, we report that the hypomethylation at non-CpG and CpG sites in the promoter around its transcription start site (TSS) is an important reason to increase HIF-1α expression in those breast cancer cells or tissue samples with highly malignant behavior. Furthermore, we provide solid evidence that DNMT3a and Mecp2 are needed for non-CpG and CpG methylation of HIF-1α in breast cancer cells. In addition, enhanced H3K9Ac modification at the promoter region which contained non-CpG and CpG sites is another reason to increase the transcription and expression of HIF-1α.

## Results

### Increased HIF-1α expression and higher HIF-1α activity in breast cancer cell lines with more malignant behaviors

**Fig. 1:**
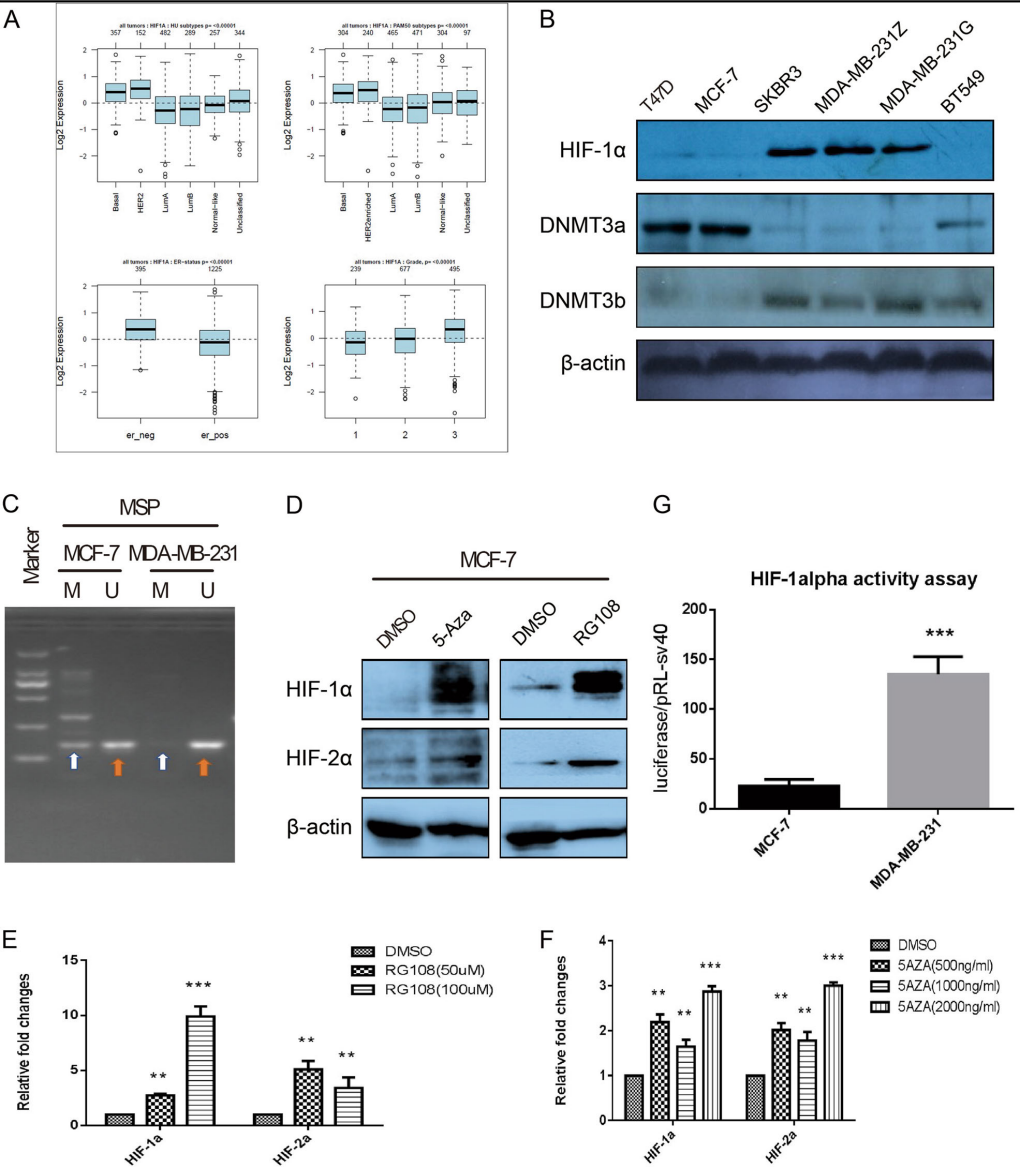
Methylation may be one of an important reason that leads to the HIF-1α downregulation in luminal subtype breast cancer cells. **a** Boxplots on the upper row showing relative expression of HIF-1α in basal, HER2 positive, luminal A, luminal B, normal-like, and nonclassified breast cancer patients. Boxplots in the lower-left corner showing relative expression of HIF-1α in ERα positive and negative status. Boxplots in the lower-right corner showing relative expression of HIF-1α in breast cancer patients with grade 1, 2, and 3. **b** Western blotting analysis of HIF-1α expression in various breast cancer cell lines. **c** MSP analysis of the methylation status of the HIF-1α promoter. “U” indicates unmethylated amplification, and “M” indicates methylated amplification. **d** Western blotting shows changes in the expression levels of HIF-1α and HIF-2α after the cells were treated with RG-108 or 5-Aza of different concentrations. **e** qRT-PCR shows changes in the expression levels of HIF-1α and HIF-2α after the cells were treated with RG-108 of different concentrations. **f** qRT-PCR shows changes in the expression levels of HIF-1α and HIF-2α after the cells were treated with 5-Aza of different concentrations. **g** HIF-1α activities in MCF-7 and MDA-MB-231 cells were tested respectively by co-transfecting 6XHRE reporter vector and a Renilla luciferase plasmid. Data are presented as the means ± SD of three independent experiments. *p < .05; **p < .01; and ***p < .001 (Student’s *t*-test) as compared to control cells

We firstly found that only basal and HER2 positive breast cancer present higher HIF-1α expression either according to HU subtype or PAM50 subtype among six distinct groups, namely, basal, HER2 positive, luminal A, luminal B, normal-like, and non-classified breast cancer, by using GOBO online analysis (co.bmc.lu.se/gobo) (*p* < 0.00001, Fig. 1a upper row). Based on ER status, we analyzed and compared expression levels and found that there is significantly higher HIF-1α mRNA expression in ER-negative than in ER-positive breast cancer (*p* < 0.00001; Fig. 1a lower-left). Additionally, statistical analysis comparing the expression levels according to tumor grade showed that there are significantly higher HIF-1α mRNA expression levels in grade III tumors than in grade I or II tumors (*p* < 0.00001, Fig. 1a lower-right). Those patients whose tumors had the lowest levels of HIF-1α expression had more favorable prognoses.

Next, the relative HIF-1α expression levels were determined by western blotting in five human breast cancer cell lines including MCF-7, T47D, SKBR3, MDA-MB-231 (two strains stored in different laboratories) and BT549, of which MCF-7 and T47D are characterized as ER-positive/PgR-positive luminal mammary carcinoma, MDA-MB-231 and BT-549 are characterized as triple-negative/basal-B mammary carcinoma (TNBC), and SKBR3 is a cell line that overexpresses the Her2 (Neu/ErbB-2) gene product. Western blotting revealed that HIF-1α expression varied among the five breast cancer cell lines. In detail, those highly invasive breast cancer cell lines, such as MDA-MB-231 and SKBR3, presented higher expression levels of HIF-1α, whereas the ER positive MCF-7 and T47D cell lines presented lower expression levels of HIF-1α (Fig. 1b).

To test whether the CpG and non-CpG methylation differentially affects HIF-1α activity in non-invasive breast cancer and invasive breast cancer cells, the luciferase activity assays were further detected in MCF-7 and MDA-MB-231 cells by co-transfecting 6XHRE reporter vector and a Renilla luciferase plasmid. The results revealed that the luciferase activity in MCF-7 was about six times higher than that in MDA-MB-231 cells (Fig. 1g; *p* < .001). Collectively, these data indicate that HIF-1α expression and activity decreased in MCF-7 breast cancer cells. Conversely, increased levels of HIF-1α expression and activity were observed in MDA-MB-231 cells or HER2 + breast cancer cells.

### Increased HIF-1α expression is associated with hypomethylation status of the HIF-1α gene promoter

To address whether promoter methylation was the cause of loss of HIF-1α gene expression, we analyzed whether there is CpG island in the promoter. The results showed that there is one large CpG island that is a cross-regulatory promoter and part of the first non-coding exon (−770bp to + 315 bp) within the HIF-1α gene (Fig. S1A). Primers for Methylation Specific PCR (MSP) were designed to target and assess the methylation status at specific CpG sites using Methyl primer Express Software v1.0 (Fig. S1B), PCR results showed that MCF-7 cell promoter possess a mixed hypermethylated and unmethylated status, whereas the HIF-1α promoter in MDA-MB-231 cells only possessed a nonmethylated status (Fig. 1c).

Furthermore,, MCF-7 cells were treated with various concentrations of 5-Aza or RG-108. Both RT-PCR and western blotting showed that HIF-1α and HIF-2α expression levels gradually increased with increasing concentrations of 5-Aza or RG-108 (Figs. 1d–f). Taken together, these data demonstrate that deceased HIF-1α expression in MCF-7 cells is probably associated with the hypermethylation status of CpG islands in its promoter.

### Hypomethylation of CpG and non-CpG sites within the HIF-1α gene promoter in breast cancer epithelial cells with highly malignant biological behavior

To elucidate which CpG sites are methylated and whether there is a difference in methylation frequency among various breast cancer cell types, bisulfite sequencing was carried out (Fig. S1C&D).

**Fig. 2:**
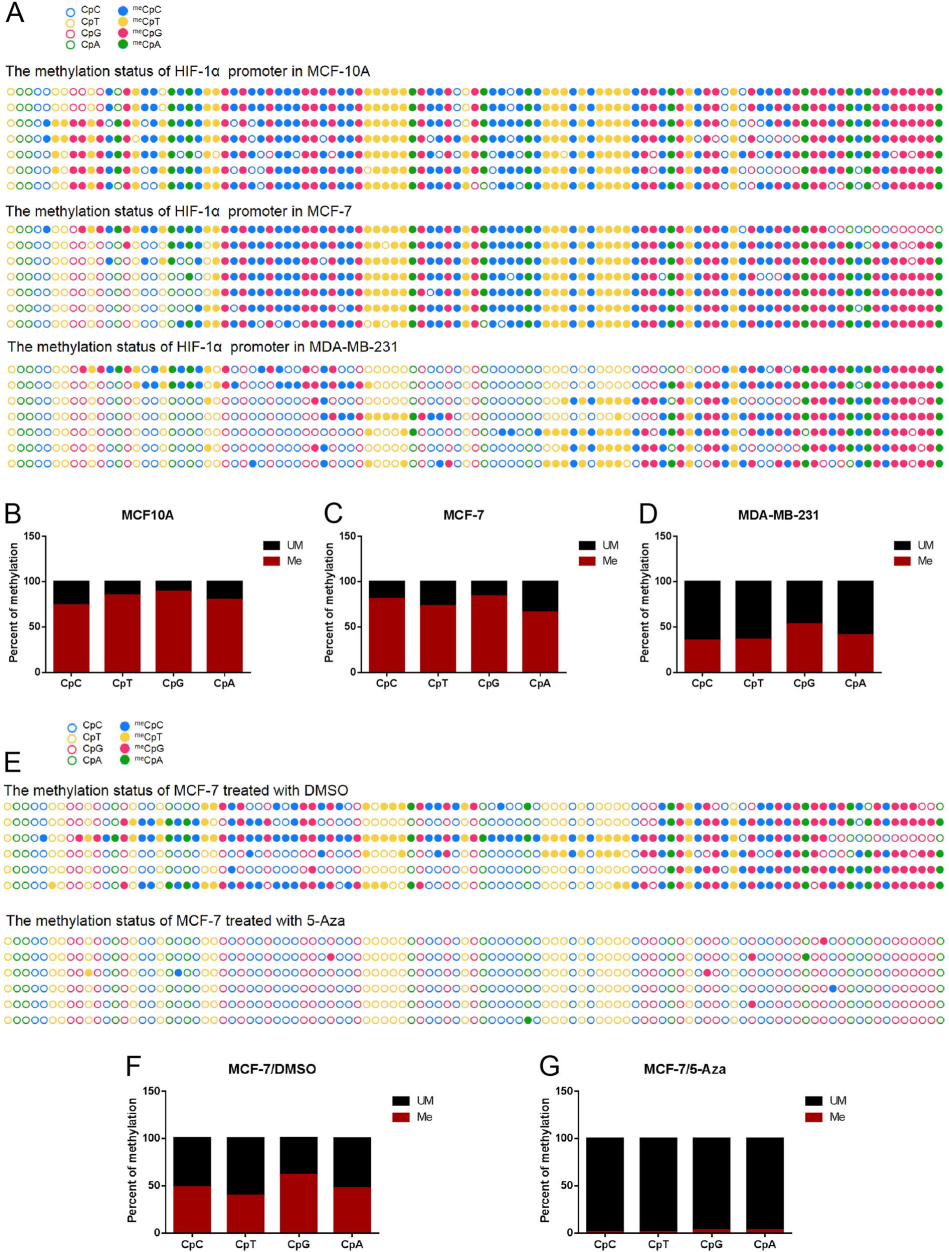
The methylation frequencies at CpG and non-CpG loci within promoter and first exon of HIF-1α gene in turn decrease among MCF-10A, MCF-7 and MDA-MB-231 cells. **a** Comparison of bisulfite sequencing results among MCF-10A, MCF-7 and MDA-MB-231 cells. Filled circles represent methylation, and blank circles represent nonmethylation. **b–d** Analyzing and comparing the methylation frequencies at CpC, CpT, CpG and CpA loci within the promoter and first exon of HIF-1α gene among MCF-10A, MCF-7 and MDA-MB-231 cells according to bisulfite sequencing results. **e** Comparison of bisulfite sequencing results between MCF-7 cells treated with DMSO and 5-Aza respectively. **f** and **g** Analyzing and comparing the methylation frequencies at CpC, CpT, CpG and CpA loci within the promoter and first exon of HIF-1α gene between MCF-7 cells treated with DMSO and 5-Aza respectively. Data are presented as the means ± SD of three independent experiments. *p < .05; **p < .01; and ***p < .001 (Student’s *t*-test) as compared to control cells

Surprisingly, when we compared and analyzed sequencing results with online software (http://quma.cdb.riken.jp/), we found that the sequence reads contain a certain amount of unconverted cytosine. By manual comparison with NCBI Blast, excepting methylation at CpG sites, we found an amount of cytosine methylation at non-CpG sites within the HIF-1α gene promoter. To analyze and display methylation status at CpG and non-CpG sites, we developed software that can recognize methylated and unmethylated cytosines at CpG and non-CpG sites. Furthermore, to ensure accurate calculation of the fidelity of DNA methylation inheritance, we strictly determined reaction conditions for all samples. First, we compared methylation status within the HIF-1α gene promoter in three different cell lines including MCF10A (benign breast epithelial cells), MCF-7 and MDA-MB-231. The sequence reads obtained were subjected to multiple alignments together with a reference sequence for the corresponding genomic locus. The results showed that MCF10A cells present with hypermethylation status (Fig. 2a upper panel), whereas hypomethylation was observed in MDA-MB-231 cells (Fig. 2a bottom panel). The methylation status of the HIF-1α gene promoter is just between these two in MCF-7 cells (Fig. 2a middle panel). In detail, the percentages of methylated CpG sites in MCF10A, MCF-7, and MDA-MB-231 were 89.40 ± 3.70% (Fig. 2b), 83.87 ± 4.56% (Fig. 2c), and 53.28 ± 9.25% (Fig. 2d), respectively.

To avoid possible omission of methylated loci, we analyzed DNA methylation at non-CpGs (CpHs) in the first exon of the HIF-1α gene in various breast cancer cell lines. The results showed the percentages of methylated CpC, CpT, and CpA sites were 74.13 ± 6.60%, 84.52 ± 6.58% and 80.22 ± 5.60%, respectively, in MCF-10A cells (Fig. 2b); 79.93 ± 6.59%, 73.21 ± 7.16% and 65.66 ± 5.48%, respectively in MCF-7 cells (Fig. 2c); and 35.13 ± 10.92%, 35.84 ± 10.23% and 40.66 ± 10.62%, respectively, in MDA-MB-231 cells (Fig. 2d). Overall, these data suggest that DNA methylation at CpG and non-CpG sites may play vital roles in HIF-1α expression during breast cancer progression.

### 5-Aza treatment significantly inhibits CpG and non-CpG methylation of HIF-1α gene promoter in MCF-7 cells

To further explore whether CpG and non CpG methylation within the HIF-1α gene promoter may determine the HIF-1α expression level in breast cancer cell lines, we detected and analyzed the CpG and non CpG methylation status of the HIF-1α promoter in MCF-7 after 5-Aza treatment (Fig. 2e). The percentages of methylated CpA, CpT, CpC and CpG were significantly decreased in MCF-7 cells treated with 5-Aza when compared with that cells treated DMSO (2.56 ± 3.97% *vs*. 47.43 ± 13.25% at CpA, *p* = 1.2495E-05; 0.70 ± 1.70% *vs*. 39.58 ± 27.23% at CpT, *p* = 0.0058; 0.90 ± 1.39% *vs*. 48.65 ± 23.19% at CpC, *p* = 0.0005; 2.69 ± 2.43% *vs*. 61.83 ± 11.79% at CpG, *p* = 2.853E-07; Figs. 2f, g). The results indicate that 5-Aza treatment can impact CpG and non CpG methylation status of the HIF-1α promoter.

### Luminal subtype human breast cancer samples present higher CpG and non-CpG methylation levels in the HIF-1α promoter as compared to triple negative breast cancer samples

**Fig. 3:**
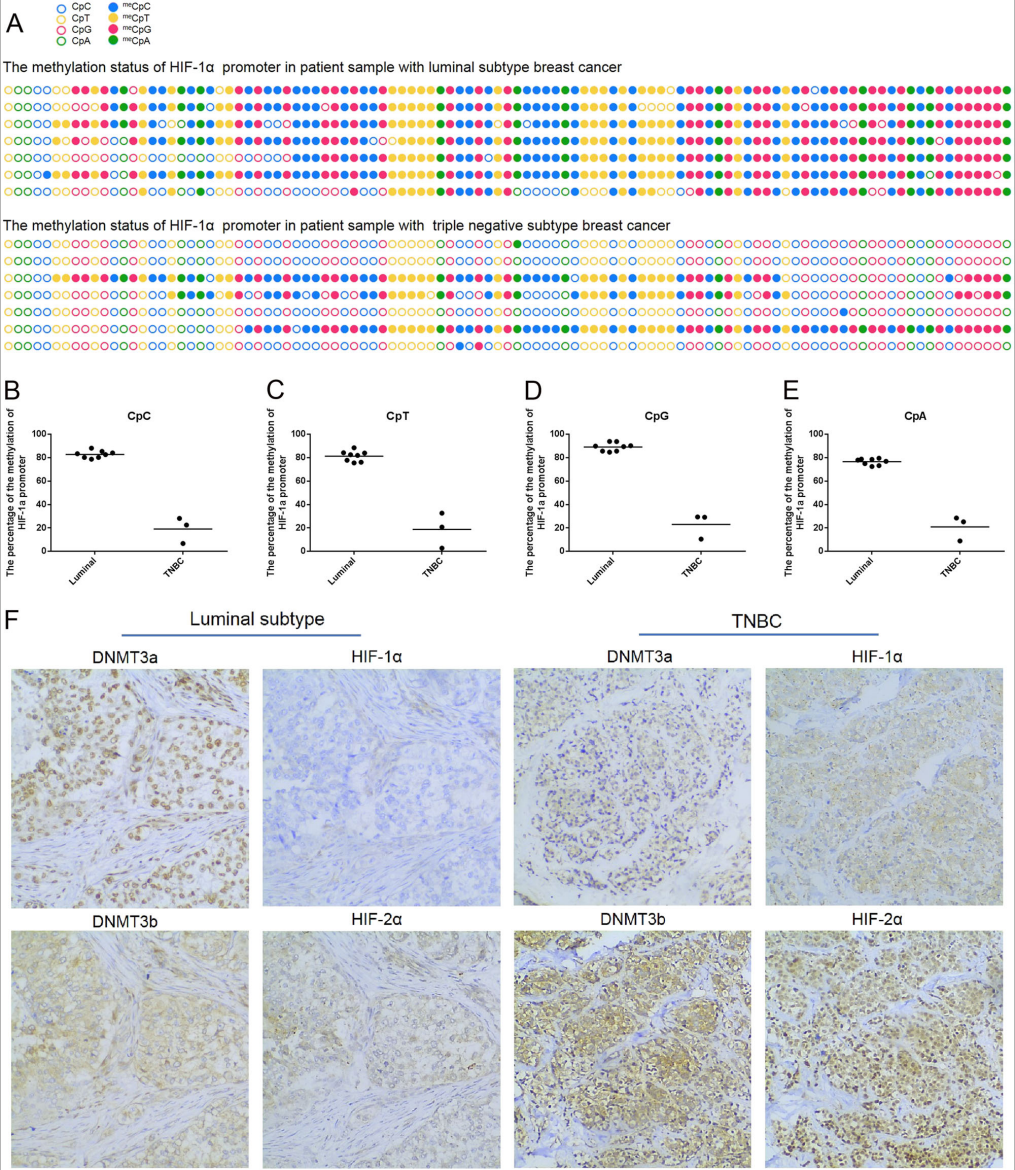
The methylation frequencies at CpG and non-CpG loci within promoter and first exon of HIF-1α gene increase in luminal subtype breast cancer tissue samples; and decrease in triple negative subtype breast cancer tissue samples. **a** Comparison of bisulfite sequencing results between luminal and TNBC subtype human breast cancer samples. Filled circles represent methylation, and blank circles represent nonmethylation. **b–e** Calculating and comparing the methylation frequencies at CpC, CpT, CpG and CpA loci within the promoter and first exon of HIF-1α gene between luminal and TNBC subtype human breast cancer samples. **f** Immunohistochemistry stain analyzing the expression DNMT3a, DNMT3b, HIF-1α and HIF-2α and their locations in total 68 cases of human breast cancer samples including 36 cases of luminal and 32 cases of TNBC subtype. Magnification, ×20. Data are presented as the means ± SD of three independent experiments. *p < .05; **p < .01; and ***p < .001 (Student’s *t*-test) as compared to control cells

DNA methylation status at CpG and non-CpGs in the HIF-1α promoter were tested in eight cases of diagnosed human luminal subtype and three cases of diagnosed triple negative breast cancer samples. Via DNA extraction and bisulfite sequencing, respectively, we found uniform hypermethylation at CpG and non-CpG sites within the promoter and first exon of the HIF-1α gene in luminal subtype breast cancer samples (Fig. 3a upper panel) and hypomethylation in TNBC samples (Fig. 3a bottom panel). More specifically, in luminal subtype breast cancer samples, the mean percentage of methylated CpC, CpT, CpG and CpA sites was 82.91 ± 3.09%, 81.39 ± 4.50%, 89.22 ± 3.59%, and 76.61 ± 2.68%, respectively; however, in TNBC samples, the mean percentage of methylated CpC, CpT, CpG and CpA sites were 19.16 ± 11.10%, 18.80 ± 15.06%, 23.06 ± 10.88%, and 20.94 ± 10.49%, respectively (Figs. 3b–e). Collectively, these data indicate that there exists hypermethylation at CpG and non-CpG sites within the first exon of the HIF-1α gene in human luminal subtype breast cancer samples as compared to TNBC samples. Furthermore, loss of methylation at CpG and non-CpG sites in the HIF-1α gene promoter may be the main reason for increased expression of HIF-1α in TNBC.

### DNMT3a may play a vital role in first exon methylation of the HIF-1α gene and inhibit DNMT3a expression

To address which methyltransferases are responsible for DNA methylation at CpG sites and non-CpGs within the first exon of the HIF-1α gene, we detected the endogenous expression levels and patterns of DNMT3a and DNMT3b in various breast cancer cell lines by western blot. As shown in Fig. 1b, DNMT3a and 3b expression levels varied among several breast cancer cell lines. In particular, DNMT3b and HIF-1α expression levels are positively correlated, with both presenting higher expression in HER2+ and TNBC cells. DNMT3a exhibited an inverse expression pattern among the five breast cancer cell lines. More specifically, DNMT3a presented the lowest expression levels, while HIF-1α exhibited higher expression levels, in HER2 + and TNBC cells. Conversely, tHIF-1α expression was barely detectable, whereas DNMT3a exhibited higher expression levels in ERα + breast cancer cell lines such as MCF-7 and T47D, suggesting that DNMT3a is most likely involved in methylation in the first exon of the HIF-1α gene in MCF-7 cells.

Because in vitro cell culture is known to affect epigenetic modification, we next sought to validate our in vitro findings in human breast cancer tissues. To confirm higher expression in TNBC samples, we first performed immunohistochemistry staining on serial slides of formalin-fixed paraffin-embedded sections from 68 human breast cancer samples, including 36 cases of luminal and 32 cases of TNBC subtype breast cancer (Fig. 3f). The expression patterns and localization of HIF-1α, HIF-2α, DNMT3a and DNMT3b were further compared and analyzed using specific antibodies. Immunohistochemistry results revealed that DNMT3a presents a higher positive rate (72.2%) in luminal subtype samples (26 positive in 36 total cases). Herein, we need to emphasize that “positive rate” refers to a much stronger DNMT3a signal localized to the nuclei in luminal subtype samples. By contrast, the positive rate was only 46.9% (15 positive in 32 total cases) in TNBC samples (*χ*^2^ = 4.546; *p* = 0.033). For the DNMT3b staining, there was a higher positive percentage in TNBC (84.4%) than in luminal samples (77.8%; *χ*^2^ = 0.477; *p* = 0.49). As shown in Table 1, we observed that the positive rate of HIF-1α in serial sections was 33.3% (12 positive in 36 total cases) in luminal subtype samples, which is significantly lower than that in TNBC subtype cases (28 positive in 32 total TNBC cases; 87.5%; *χ*^2^ = 20.521; *p* < 0.0001). Similarly, HIF-2α exhibits a higher positive rate TNBC subtype breast cancer (26 positive in 32 total cases; 81.2%) than luminal subtype breast cancer (20 positive in 36 total cases; 55.6%; *χ*^2^ = 5.110; *p* = 0.024). Taken together, these results suggest that DNMT3a may be the primary protein responsible for methylation of CpG and non-CpGs sites in the HIF-1α gene promoter around TSS and, thus, suppresses HIF-1α expression in luminal breast cancer.

**Table 1:** Correlation analysis among DNMT3a, DNMT3b, HIF-1α and HIF-2α protein expression in luminal and triple negative breast cancer samples

protein	Total (*n*)	luminal			TNBC			*χ* test
		Positive *N* (%)	Negative *N* (%)	*n*	Positive *N* (%)	Negative *N* (%)	*n*	
DNMT3a	68	26(72.2%)	10(27.8%)	36	15(46.9%)	17(53.1%)	32	*χ*^2^ = 4.546 *p* = 0.033
HIF-1a	68	12(33.3%)	24(66.7%)	36	28(87.5%)	4(12.5%)	32	*χ*^2^ = 20.521 *p* < 0.0001
DNMT3b	68	28(77.8%)	8(22.2%)	36	27(84.4%)	5(15.6%)	32	*χ*^2^ = 0.477 *p* = 0.49
HIF-2a	68	20(55.6%)	16(44.4%)	36	26(81.2%)	6(18.8%)	32	*χ*^2^ = 5.110 *p* = 0.024

### Increased DNMT3a expression is involved in hypermethylation at CpG and non-CpG sites in the promoter of the HIF-1α gene

**Fig. 4:**
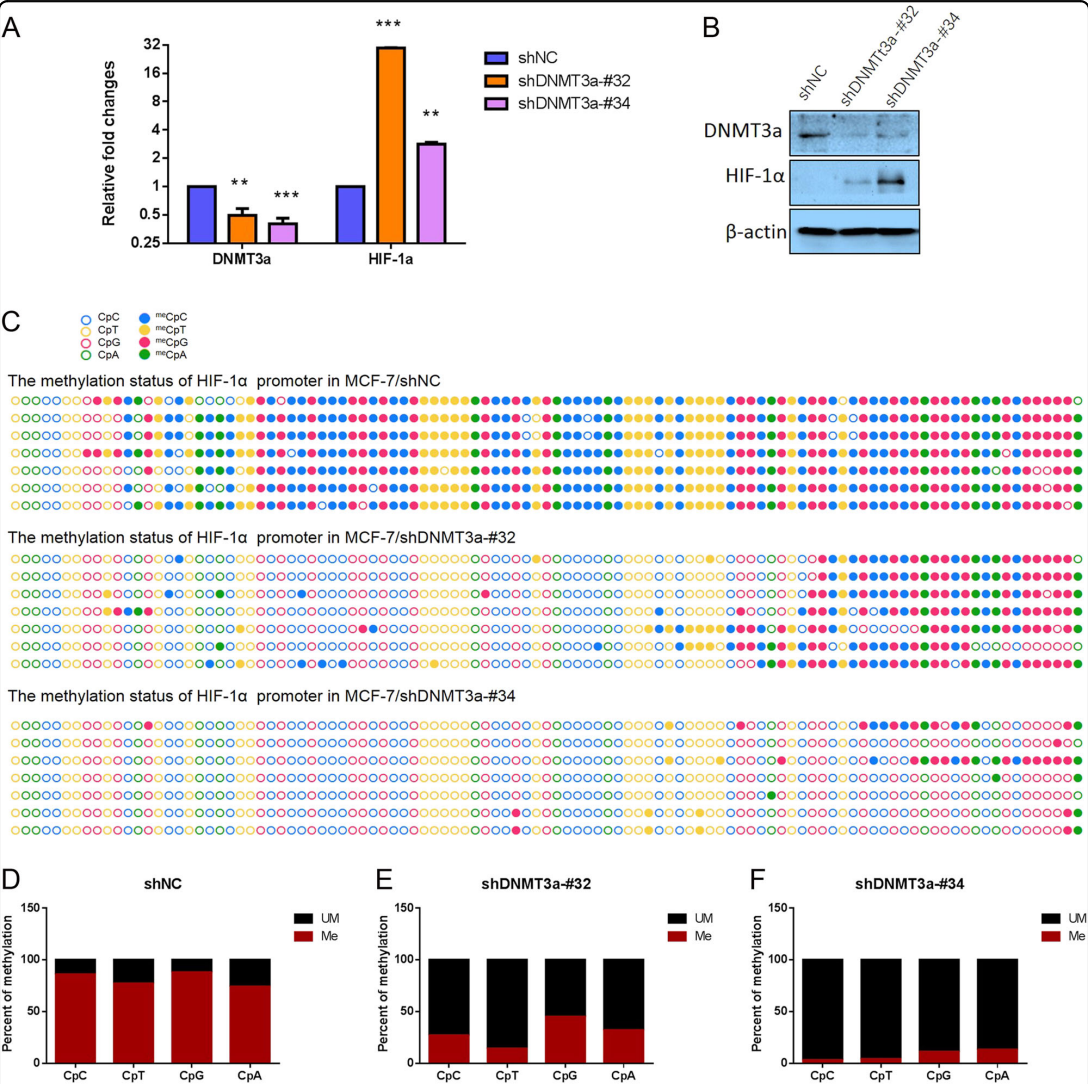
Identifying that DNMT3a involves in the methylation at CpC and non-CpC loci within promoter and first exon of HIF-1α gene by knocking down DNMT3a. **a** RT-PCR shows that the expression of HIF-1α is upregulated after DNMT3a knockdown. **b** Western blotting revealed that the expression of HIF-1α is upregulated after DNMT3a knockdown. **c** Comparison of bisulfite sequencing results after DNMT3a knockdown (containing two target sites) in MCF-7 cells. Filled circles represent methylation, and blank circles represent nonmethylation. **d–f** Calculating and comparing the methylation frequencies at CpC, CpT, CpG and CpA loci within the promoter and first exon of HIF-1α gene after DNMT3a knockdown. Data are presented as the means ± SD of three independent experiments. *p < .05; **p < .01; and ***p < .001 (Student’s *t*-test) as compared to control cells

**Fig. 5:**
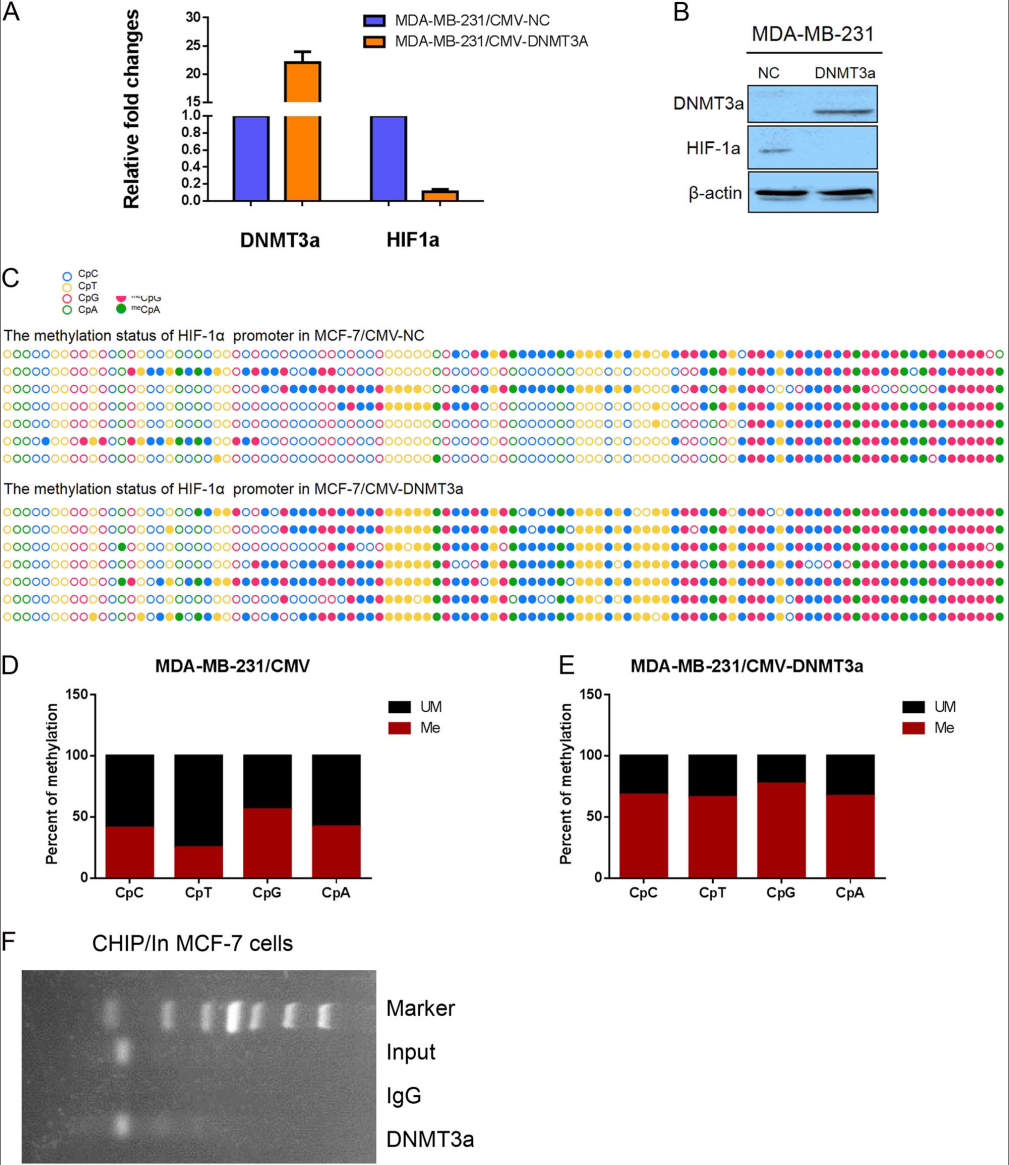
Identifying that DNMT3a involves in the methylation at CpC and non-CpC loci within promoter and first exon of HIF-1α gene by overexpressing DNMT3a. **a** RT-PCR shows that the expression of HIF-1α is downregulated after overexpressing DNMT3a. **b** Western blotting revealed that the expression of HIF-1α is downregulated after overexpressing DNMT3a. **c** Comparison of bisulfite sequencing results after overexpressing DNMT3a in MDA-MB-231 cells. Filled circles represent methylation, and blank circles represent nonmethylation. **d–e** Calculating and comparing the methylation frequencies at CpC, CpT, CpG and CpA loci within the promoter and first exon of HIF-1α gene after overexpressing DNMT3a. Data are presented as the means ± SD of three independent experiments. *p < .05; **p < .01; and ***p < .001 (Student’s *t*-test) as compared to control cells

To further confirm the hyposthesis that upregulated DNMT3a is involved in hypermethylation at CpG and non-CpG sites within HIF-1α gene promoter, we established an MDA-MB-231 cell strain stably overexpresing DNMT3a (Fig. S2A&B) and an MCF-7 cell strain with stable knockdown of DNMT3a (Fig. S2C&D) using the lentiviral vector systems. DNMT3a downregulation-orup, respectively, in MCF-7/shDNMT3a-#32 and -#34 (Figs. 4a, b) or MDA-MB-231/CMV-DNMT3a (Figs. 5a, b) were confirmed by qRT-PCR and western blotting. Importantly, HIF-1α expression was up - or downregulated at the mRNA and protein levels along with DNMT3a expression changes in MCF-7/shDNMT3a-#32 and -#34 or MDA-MB-231/CMV-DNMT3a cells, respectively, suggesting that HIF-1α is indeed inhibited by DNMT3a through methylation (Figs. 4a, b, Figs. 5a, b). To elucidate whether CpG or non-CpG sites, and, further, which non-CpG sites within the first exon of the HIF-1α gene are methylated by DNMT3a, bisulfite sequencing was performed. The results revealed that in MCF-7/shDNMT3a-#32 and -#34 cells, the proportions of methylated CpC, CpT, CpG and CpA sites were 26.75 ± 4.28% and 2.76 ± 4.33%, 14.36 ± 10.97% and 4.17 ± 3.86%, 44.70 ± 6.02% and 11.06 ± 12.89%, and 31.87 ± 6.92% and 13.19 ± 9.83%, respectively, which were significantly lower than those in MCF-7/shNC cells (86.10 ± 1.87% at CpC sites, 77.38 ± 6.74% at CpT sites, 88.02 ± 5.50% at CpG sites, and 74.73 ± 8.56% at CpA sites; Figs. 4c–f; All *p* values were less than 0.001). Conversely, the proportions of methylated CpC, CpT, CpG and CpA sites were 67.67 ± 10.56%, 66.36 ± 9.65%, 77.42 ± 8.12%, and 67.03 ± 8.56%, respectively, in MDA-MB-231/CMV-DNMT3a, which were significantly higher than those in MDA-MB-231/CMV, (41.31 ± 14.36%, 24.99 ± 15.40%, 56.50 ± 9.00%, and 41.76 ± 8.72%, respectively; all *p* values were less than 0.001; Figs. 5c–e).

To elucidate whether DNMT3a can bind to CpG or non-CpG regions of the HIF-1α promoter, we also performed CHIP assay using MCF-7 cells. In detail, anti-DNMT3a antibody was used to identify DNMT3a binding the CpG or non-CpG regions of the HIF-1α promoter, while “input” was used as a positive control and non-specific IgG was used as a negative control. The results showed that DNMT3a can bind to the region containing CpG and non-CpG sites in the HIF-1α promoter (Fig. 5f).

To further exclude effects of DNMT1 or DNMT3b on methylation at CpG and non-CpG sites within HIF-1α gene promoter across TSS, MCF-7 cell strains with stable knockdown of DNMT1 or DNMT3b were established using a lentiviral vector system with GFP (Fig. S3A&B). According to qRT-PCR analysis, HIF-1α mRNA levels did not appreciably change when DNMT1 or DNMT3b were knocked down as compared to control cells (Fig. S3C&D). DNMT1 and DNMT3b knockdown efficiencies were confirmed by western blotting (Fig. S3E, F&G). Moreover, bisulfite sequencing results showed no significant differences in the proportion of CpA, CpC, CpG or CpT site methylation between MCF-7/shNC and MCF-7/shDNMT1 (Fig. S4) or MCF-7/shNC and MCF-7/shDNMT3b lines (Fig. S5). In summary, our data demonstrate that DNMT3a plays an important role in hypermethylation at CpG and non-CpG sites in MCF-7 cells and luminal subtype breast cancer tissue.

### Increased Mecp2 expression enhances methylation at CpG and non-CpG sites in the promoter of the HIF-1α gene

**Fig. 6:**
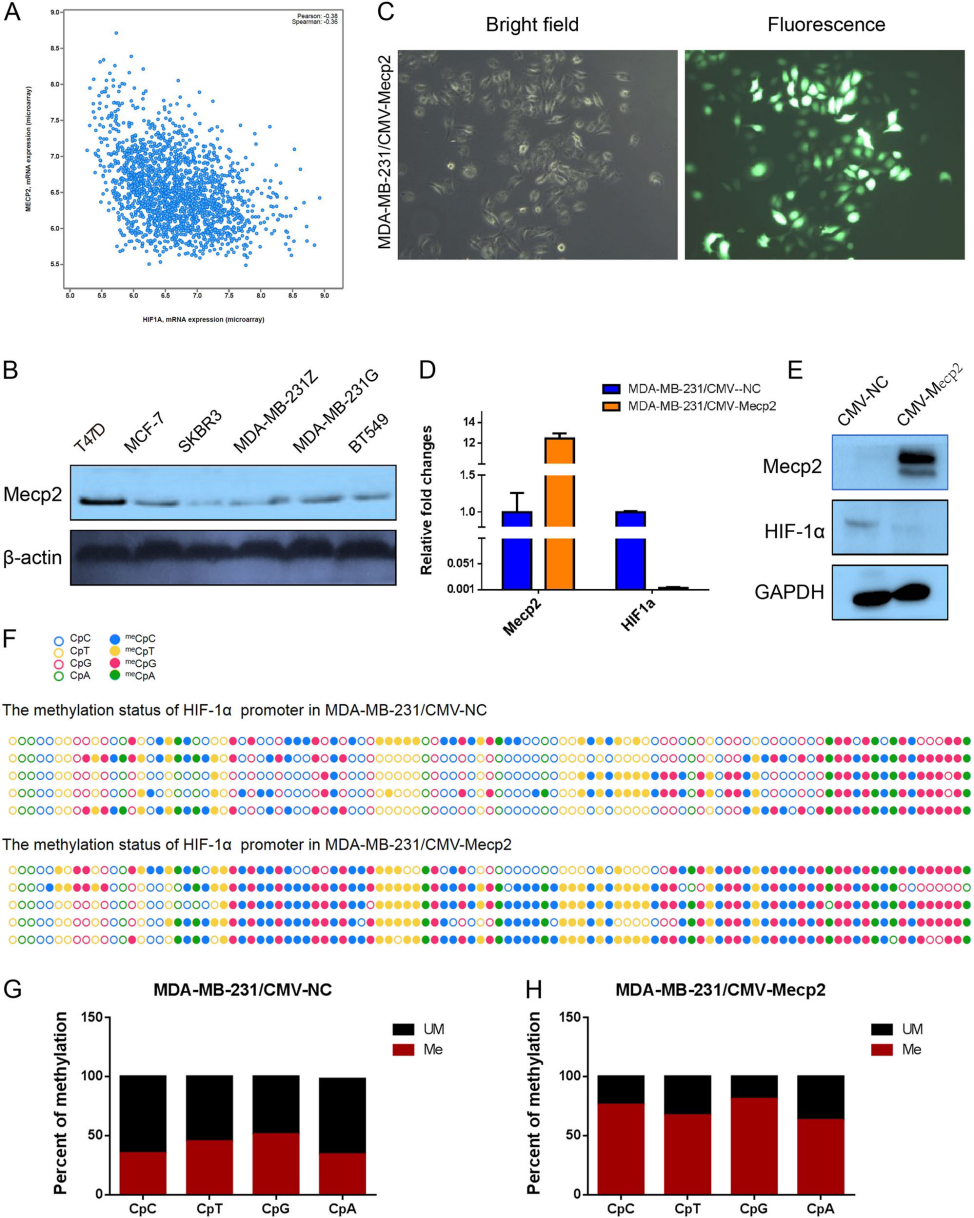
Mecp2 involve in the non-CpG methylation within HIF-1α promoter in MDA-MB-231 cells. **a** Negative correlation between MECP2 and HIF-1α expression exists in human breast cancer tissues. Correlation of MECP2 mRNA and HIF-1α levels in breast cancer patients are directly obtained from the online data (www.cbioportal.org). **b** Western blotting analyzed the expression level of Mecp2 in various breast cell lines. **c** Establishing the stable overexpression-Mecp2 cell line in MDA-MB-231 cells. **d** Validating the efficiency of Mecp2 overexpression and its effect on Notch3 expression level by qRT-PCR analysis. **e** Validating the efficiency of Mecp2 overexpression and its effect on HIF-1α expression level by western blotting analysis. **f** Comparison of bisulfite sequencing results after overexpressing Mecp2 in MDA-MB-231 cells. Filled circles represent methylation, and blank circles represent nonmethylation. **g** and **h** Comparison of the percentages of methylated CpG and CpH within HIF-1α promoter from MDA-MB-231 cells before and after Mecp2 overexpression. Data are presented as the means ± SD of three independent experiments. *p < .05; **p < .01; and ***p < .001 (Student’s *t*-test) as compared to control cells

Furthermore, we analyzed whether an increase in HIF-1α mRNA expression is associated with a decrease in Mecp2 levels in the same set of human breast cancer samples. We found that the mRNA levels of both molecules were significantly negatively correlated (Pearson correlation coefficient = 0.496, *p* = 0.003; Fig. 6a). As shown in Fig. 6b, according to endogenous Mecp2 and HIF-1α protein expression levels and patterns in various breast cancer cell lines, we noted that Mecp2 expression varied among several breast cancer cell lines and presented a reverse correlation to that of HIF-1α. To elucidate whether MeCp2 plays a role in hypermethylation at CpG and non-CpG sites in the first exon of the HIF-1α gene, an MDA-MB-231 stable cell strain overexpressing MeCp2 was established using a lentiviral system (Fig. 6c); overexpression efficiency was confirmed by RT-PCR and western blotting. As shown in Figs. 6d, e, overexpressing MeCp2 significantly inhibited HIF-1α expression in MDA-MB-231 cells as compared to control cells. Bisulfite sequencing was also performed(Fig. 6f). The proportions of methylated CpC, CpT, CpG and CpA sites were 75.68 ± 11.62%, 66.67 ± 14.13%, 80.64 ± 6.04%, and 63.08 ± 10.03%, respectively, in MDA-MB-231/CMV-Mecp2, which were significantly higher than those in MDA-MB-231/CMV, which were 34.60 ± 6.17%, 34.05 ± 11.65%, 51.10 ± 9.61%, and 44.62 ± 10.03%, respectively (All *p* values were less than 0.001; Figs. 6g, h).

### CpG and non-CpG methylation results in a substantial decrease in luciferase activity

**Fig. 7:**
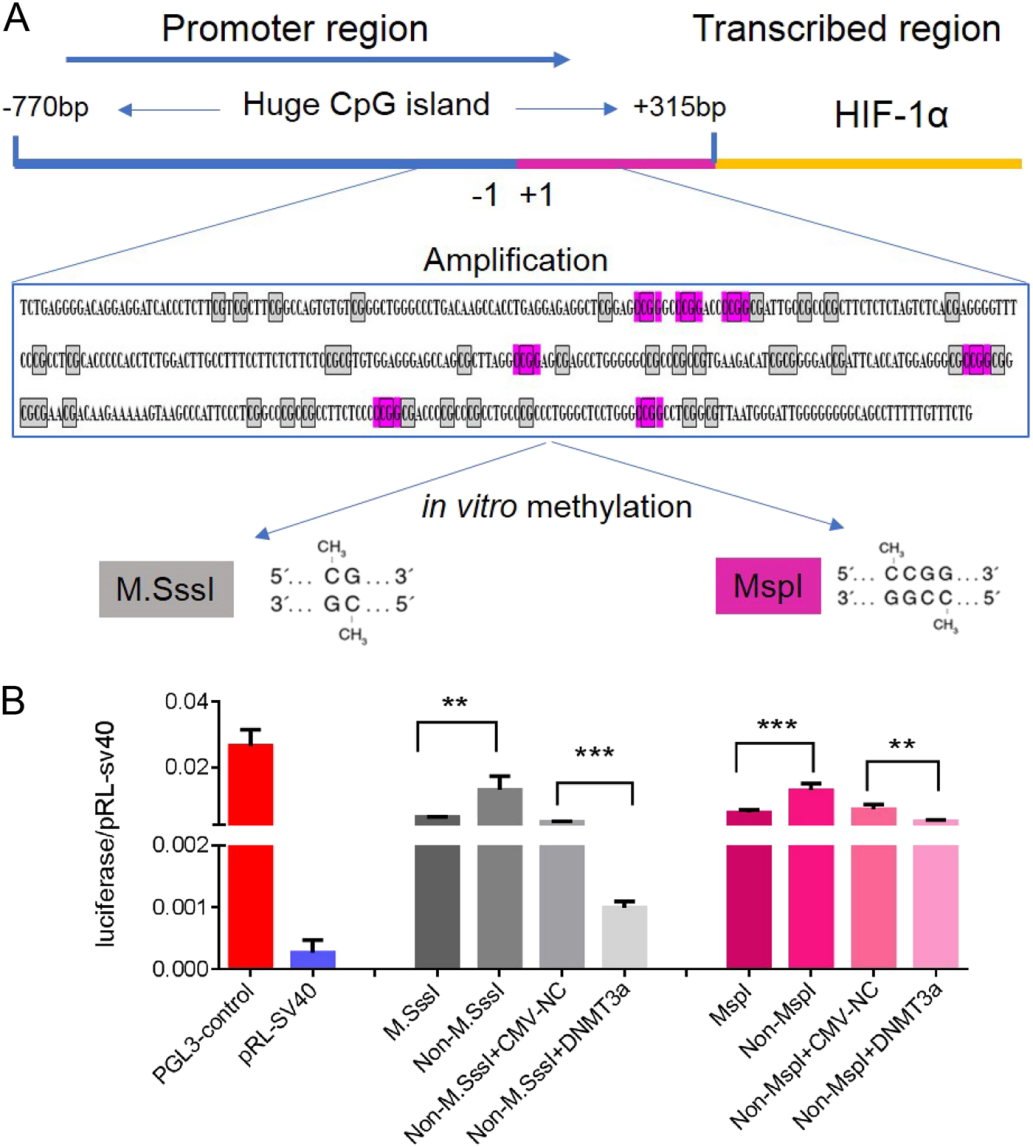
Non-CpHs methylation can silence HIF-1α gene transcription. **a** Analyzing in vitro DNA-methyltransferase, MSP I and MSss I recognition sites within HIF-1α gene promoter. **b** The methylation at CpC and CpG sites could cause transcriptional repression of HIF-1α gene by using luciferase activity assay. Data are presented as the means ± SD of three independent experiments. *p < .05; **p < .01; and ***p < .001 (Student’s *t*-test) as compared to control cells

To test whether transcriptional repression of the HIF-1α gene is directly affected by CpG and non-CpG methylation, we performed in vitro methylation using MSPI and MSssI (Fig. 7a) and luciferase activity assays. Successful in vitro methylation was validated by methylation-sensitive endonuclease restriction (Fig. S1E). As shown in Fig. 7b, luciferase activity was significantly downregulated in cells transfected with reporter vector containing PCR product treated with MSPI or MSssI as compared to control cells (fragments not treated with MSPI or MSssI). When DNMT3a overexpression vector was also transfected into 293 T cells, luciferase activity was further reduced as compared to cells transfected with empty vector. Collectively, these findings suggest that CpG and non-CpG methylation within the HIF-1α gene promoter and first exon may play key roles in HIF-1α expression in breast cancer, and that this methylation at CpG and non-CpG sites is regulated by DNMT3a.

### H3K9ac can bind to the region of the HIF-1α gene containing CpG and non-CpG sites to activate transcription

**Fig. 8:**
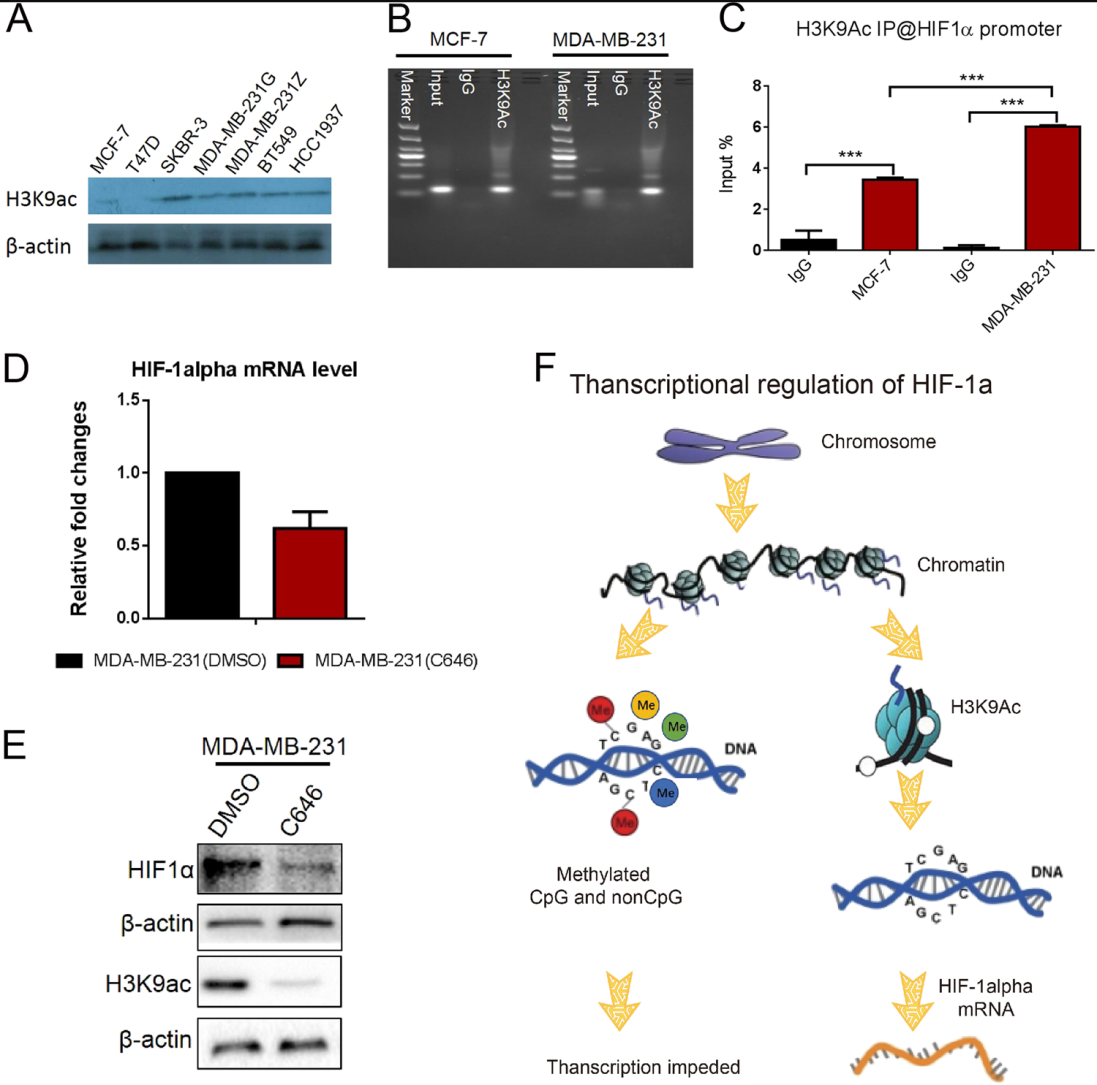
Two epigenetic mechanisms, DNA hypomethylation and H3K9ac histone modifications, together activate HIF-1α gene in TNBC breast cancer cells and tissue samples. **a** Western blotting analysis of H3K9ac expression level in various human breast cell lines. **b** Normal IgG or anti-H3K9ac antibodies were used in a ChIP assay to determine that H3K9ac binds in the HIF-1α promoter in breast cancer cells. **c** ChIP-qPCR analysis of H3K9ac recruitment on HIF-1α promoter in MDA-MB-231 cells and MCF-7 cells. The ChIP-qPCR results represent % of input chromatin. **d** The analysis of transcriptional level of HIF-1α in MDA-MB-231 breast cancer cell line treated with C646 or DMSO. **e** The western blotting analysis of H3K9ac and HIF-1α proteins expression in MDA-MB-231 breast cancer cell line treated with C646 or DMSO. **f** A working model for regulating the transcription of HIF-1α gene by the methylation at non-CpG and CpG sites and H3K9ac modification. Data are presented as the means ± SD of three independent experiments. *p < .05; **p < .01; and ***p < .001 (Student’s *t*-test) as compared to control cells

To elucidate which histone regulates HIF-1α expression, we detected expression of a panel of histone markers by western blotting. The results show that only H3K9ac expression is upregulated and parallels that of HIF-1α in those breast cancer cell lines with highly malignant behavior (Fig. 8a), suggesting that H3K9ac may activate HIF-1α gene transcription. To answer this question, chromatin immunoprecipitation (ChIP) assays were performed in MCF-7 and MDA-MB-231 cells. Anti-H3K9ac antibody was used to identify binding between H3K9ac and CpG or non-CpG regions of the HIF-1α promoter, while “input” was used as a positive control and non-specific IgG was used as a negative control. The results show that H3K9ac binds to the region containing CpG and non-CpG sites (Fig. 8b). This result is supported by UCSC browser (Fig. S5D). ChIP-quantitative PCR (qPCR) was then performed in MCF7 and MDA-MB-231 cell lines. The amount of H3K9ac binding to the region in the HIF-1α promoter containing CpG/non-CpG sites was markedly lower in MCF7 than in MDA-MB-231 cells (Fig. 8c). To show that H3K9ac may contribute to activate HIF-1α transcription, we also treated the cells with C646. The results showed that the HIF-1α transcription level in those MDA-MB-231 cells treated with C646 decreased when compared with control cells after 24 h of incubation (Fig. 8d). The protein levels of histone H3K9 acetylation and HIF-1α were lower in MDA-MB-231 cells treated with C646 when compared with control cells (Fig. 8e). Taken together, these results reveal that H3K9ac may contribute to activate HIF-1α transcription by binding to this region containing CpG/non-CpG sites surrounding the TSS.

## Discussion

Previous studies have reported that HIF-1α activity is induced by hypoxia in almost all cell types. However, under certain circumstances in tumor, HIF-1α can accumulate under normoxic conditions, promoting angiogenesis and cancer progression^16,17^. In the present study, we found the expression levels of HIF-1α in MDA-MB-231 cells and TNBC patient samples are significantly higher than that in benign or luminal epithelial cells and tissue samples. Likewise, the GOBO database also shows that HIF-1α expression levels in basal-like and HER2 + subtypes are significantly higher other breast cancer subtypes. and the expression of HIF-1α negatively correlates to ERα status and positively correlates to tumor grade.

In reviewing the literature and analyzing the structure of the HIF-1α gene, we found one large CpG island is present around the TSS. However, the studies related to methylation of the HIF-1α gene promoter are relatively rare. Pawel et al. assessed the methylation status of the HIF-1α promoter region; however, they did not find any difference in DNA methylation between control or uterine cervical carcinoma tissue samples^18^ Luo and Wang reported that the lysine methyltransferases G9a and GLP directly bind to HIF-1α and catalyze mono- and di-methylation of HIF-1α at lysine (K) 674 in vitro and in vivo^19^, they did not examine DNA methylation. A recent study suggested that autoregulation of HIF-1α at the transcriptional level occurs in primary colon cancer specimens^20^.

We therefore hypothesized that the methylation status of the HIF-1α promoter may be one of reasons to determine its expression levels in different subtypes of breast cancer cell lines and tissue samples. As expected, 5-Aza or RG-108 treatment significantly increased HIF-1α expression at transcriptional and protein levels in MCF-7 cells. Also, both methylated and unmethylated PCR products were observed in MCF-7 cells, whereas only unmethylated PCR products were found in MDA-MB-231 cells. Furthermore, cBioportal data (TCGA, Cells (2015)) show significant negative correlations between HIF-1α promoter methylation and mRNA expression level in 107 basal-like breast cancer samples (Fig. S1F), as well as between Mecp2 and HIF-1α mRNA expression levels. These data suggest that decreased HIF-1α expression in MCF-7 cells results from a methylated CpG island within the HIF-1α promoter.

An unexpected finding is the methylation of cytosines in the context of CpA, CpT, and CpC (non-CpG methylation) sites. Although it has been reported for decades, a number of studies have shown that non-CpG methylation is common in plant^21^, bacteria^22,23^ and yeast^24^. Non-CpG methylation in mammals is quite rare and is mainly restricted to specific cell types, such as pluripotent stem cells^25–30^ and oocytes^31–33^. Recently, as our understanding of these events has increased, it has been demonstrated that the non-CpG methylation is not restricted to pluripotent cells; for example, non-CpG methylation can be particularly abundant in mouse and human brain tissue, which provides theoretical evidence for us to further study non-CpG methylation within the HIF-1α gene promoter in breast cancer cells. Hence, we proposed that non-CpG hypermethylation in MCF-7 cells might be an important means of silencing HIF-1α gene transcription.

Hypermethylation or hypomethylation of non-CpG sites within the HIF-1α promoter around the TSS have not previously been described in breast cancer. In this study, we found that non-CpG methylation in the HIF-1α promoter has several characteristics. First, non-CpG methylation sites are located within the CpG island in the HIF-1α promoter around the TSS, suggesting that non-CpG and CpG methylation coexist. We speculate that there are two possible explanations for this phenomenon. One is that the addition of further methyl groups to DNA by reiterative de novo methylation at CpC, CpT and CpA sites could enhance the efficiency of transcriptional repression. Alternately, breast cancer cells employ rapid epigenetic reprogramming and, thus, induce de novo methylation at CpC, CpT and CpA sites. Second, we observed gradual increases in methylation frequencies at both non-CpG and CpG loci with increasing malignant features from the benign, luminal subtype to TNBC breast epithelial cells and tissue samples. Current knowledge regarding non-CpG methylation of DNA, including prevalence, role(s), regulation and epigenetic function(s), is still in its infancy. Thus, discerning the true implications of non-CpG methylation of DNA remains difficult. Previous studies suggest that non-CpG methylation is a by-product of hyperactive non-specific de novo methylation at CpG sites^29,34^. Growing evidence suggests that non-CpG methylation is correlated with gene expression and tissue specificity^35^.

DNMTs are enzymes involved in the transfer of methyl groups to cytosines in DNA. Dnmt1, Dnmt3a, and Dnmt3b are DNMT family members. Dnmt1 is essential for the maintenance of methylation and chromatin stability^36^, and Dnmt3a and Dnmt3b act as de novo methyltransferases, and are important for DNA methylation in the early embryonic stages^37^. Furthermore, studies demonstrated that Non-CpG methylation is carried out by the de novo methyltransferases DNMT3a and DNMT3b, while the maintenance methyltransferase DNMT1 is not associated with non-CpG methylation patterns^38^. The published study showed that mouse germinal vesicle oocytes (GVOs) lacking Dnmt3a or Dnmt3L, which works in association with DNMT3, show global reductions in both non-CpG and CpG methylation^39^. In addition, similar to 5mCs in CpGs, non-CpG methylation can also be recognized by MeCP2 (methyl-CpG binding protein 2)^40^. Zoghbi and colleagues showed that MeCP2 binds methylated non-CpG with higher affinity than nonmethylated identical DNA sequences to influence the transcriptional level of some genes, such as Bdnf gene, in the adult brain.

We therefore focused our attention on two main questions. The first question is whether there is a common underlying mechanism at work for methylation at non-CpG sites in the HIF-1α promoter in breast cancer cells. We found that there was an inverse correlation between the HIF-1α and DNMT3a expression levels in breast cancer cell lines and patient samples, suggesting that DNMT3a may be responsible for methylation of the HIF-1α promoter. This conclusion is further supported by several observations.① Overexpression or knockdown of DNMT3a expression leads to decreases or increases in methylation frequency at non-CpG loci within the HIF-1α promoter, respectively, in MDA-MB-231 and MCF-7 cells. ②We found that once DNMT3a was efficiently knocked down, the mRNA level of HIF-1alpha increased in non-invasive MCF-7 breast cancer cells. Accordingly, the protein level of HIF-1alpha increased too. ③DNMT3a can bind to the region containing CpG and non-CpG sites in the HIF-1α promoter. ④ Unlike with DNMT3a, overexpressing or knocking down DNMT3b or DNMT1 expression in MDA-MB-231 or MCF-7 cells does not lead to corresponding variation in methylation frequency at non-CpG loci in the HIF-1α promoter. These data demonstrate that DNMT3a knockdown decreases the methylation level of CpG and non-CpG islands in the promoter of HIF-1alpha around TSS, which will increase the transcription of HIF-1α gene and the expression of HIF-1α protein. In another word, although HIF-1alpha is primary regulated at the post-translational levels, there is transcriptional regulation of HIF-1alpha in breast cancer cells, such as the methylation at CpG and non-CpG dinucleotides in the HIF-1α promoter around the TSS which is mainly mediated by DNMT3a.. Besides our study, published study has shown that DNMT3a methylates and silences EPAS1 in normal cells, and DNMT3a epigenetic program is a gatekeeper of the hypoxic cancer cell phenotype^41^.

In addition, we further found that a well-characterized CpG methylation reader, Methyl-CpG-binding protein 2 (MeCP2), can read not only non-CpG methylation, but can also enhance methylation at non-CpG sites and lead to transcriptional repression of the HIF-1α gene in breast cancer cells.

The second issue that we focused on is whether non-CpG hypermethylation within the HIF-1α promoter around the TSS contributes to transcriptional repression of the HIF-1α gene. We noticed that non-CpG sites are also located in the promoter and first exon overlapping the CpG region. A prior study has shown that CpG island methylation in the first exon is tightly linked to transcriptional silencing^42^. To assess whether methylation at non-CpG sites within the HIF-1α promoter around the TSS suppress HIF-1α expression, we utilized a pGL3-enhancer luciferase gene-containing vector to perform luciferase reporter assays. To avoid the possible existence of CpG sites in the coding region of the luciferase gene and vector backbone, we employed patch methylation, which treats PCR fragments with (or without) in vitro methyltransferase. Limited by types of in vitro methyltransferases and their recognition sites, we chose to employ MspI, and MSssI. As expected, luciferase assay analyses demonstrated that methylation at both non-CpG and CpG loci within the promoter around the TSS can directly silence HIF-1α gene expression in MCF-7 breast cancer cells.

Although different views regarding the roles of methylation at non-CpG sites in regulating gene expression exist^43^, our finding is consistent with prior reports^44^. Indeed, studies have described select genes involved in mitochondrial function and fuel utilization, such as Sry^45^, PGC-1α^46,47^, PDK4, and PPAR-δ^48,49^ that are regulated by non-CpG methylation.

Another unanticipated discovery of our study is the direct crosstalk between H3K9ac and non-CpG/CpG region of HIF-1α promote. It is well known that the active H3K9ac and repressive polycomb EZH2-associated H3K27Me3 are closely associated with active or inactive gene transcription^50^. There is little evidence for a relationship between H3K9Ac levels and non-CpG methylation, especially in breast cancer cells, but in this study, we noted that H3K9ac level inversely correlate to methylated cytosine content, namely, lower level of H3K9ac is found in the non-CpG/CpG region around the TSS of HIF-1α promote in MCF-7 cells, and higher level are found in MDA-MB-231 cells. Very interesting, C646, a specific inhibitor that selectively blocks HATS of the p300/CBP family, can reduce histone H3 acetylation^51,52^. In this study, we found that C646 significantly downregulated the transcription of HIF-1α and the protein expression levels of H3K9 acetylation and HIF-1α in the MDA-MB-231 cells. Numerous facts suggest that increased H3K9Ac modification promotes HIF-1α transcription and expression in breast cancer cells.

For the breast cancer cell lines, to our surprise, unlike SKBR3 and MDA-MB-231 cell lines, the triple-negative BT549 cells don’t express HIF-1α. We speculated that the possible reason should be due to the following aspects. On the one hand, although both BT549 and MDA-MB-231 are TNBC cells, TNBC is an aggressive subtype characterized by extensive intercellular heterogeneity of gene expression programs within each tumor. The sources of BT549 and MDA-MB-231 are different. The former is primary breast cancer and the latter is isolated from pleural effusion. On the other hand, some mechanisms may contribute to altered HIF-1α protein expression. For example, it is well known that HIF-1α is primary regulated at the post-translational levels, such as VHL-dependent HIF-1α ubiquitination and degradation under normoxic conditions^53,54^, the mechanism underlying normoxic HIF1α stabilization in TNBC remains elusive. In the future research, we may focus on Why BT549 cells don’t express HIF-1α.

Herein, by combining previously published data and our findings, we have constructed a working model for epigenetic regulation of the HIF-1α at transcription level in breast cancer (Fig. 8f), and proposed that the coordinated effects of lower methylation frequencies at non-CpG/CpG sites and enhanced H3K9ac modification contribute to maintain the higher HIF-1α expression levels, particularly in breast cancer cells and tissue sample with highly malignant behaviors.

Taken together, we report that non-CpG and CpG hypermethylation occurring within the HIF-1α gene promoter around the TSS in MCF-7 cells and luminal subtype breast cancer samples is an important mediator of reduced HIF-1α expression. Conversely, in MDA-MB-231 cells and TNBC tissue samples, non-CpG loci of the HIF-1α gene are in hypomethylated status. Furthermore, these non-CpG sites are strongly associated with H3K9ac, which may establish a tissue-specific epigenetic modification pattern for HIF-1α gene transcriptional regulation, pointing to new directions for future understanding of this epigenetic modification in breast cancer therapy.

Future studies should probe the functions of non-CpG methylation during the development and progression of breast cancer.

## Materials and methods

### Human breast cancer samples, ethics approval and consent to participate

Human breast cancer specimens were obtained from 79 patients who underwent breast cancer surgery at the Cancer Hospital of Shantou University Medical College, China between 2013 and 2014. This study was approved by the Shantou University Medical Cancer Hospital Research Ethics Committee, and was performed in accordance with the Code of Ethics of the World Medical Association (Declaration of Helsinki). Written informed consent was obtained from each patient.

### Cell lines, antibodies, and reagents

All cells were cultured as recommended by ATCC. The cells were subjected to treatment with recombinant proteins and small interfering RNA (siRNA)-mediated gene knockdown. Quantitative real-time PCR, western blotting were performed and described in the Supplementary Methods section

### Vectors, transient transfection, and stable cell line construction

Standard transfection protocol was performed and described in the Supplementary Methods section.

### CpG island predictions, MSP and BSP primer design, and DNA extraction

Methyl Primer Express Software v. 1.0 was used to analyze the HIF-1α gene promoter and to design MSP and BSP primers. Genomic DNA was extracted from breast cancer cell lines and tissue samples using standard methods. A TIANamp Genomic DNA Kit (Cat. #DP304-03; TIANGEN Biotech Co., Beijing, China) was used according to the manufacturer’s instructions.

### Bisulfite sequencing

Bisulfite sequencing was performed at BBILife Sciences Corporation (Shanghai, China). The detailed processes were described in supplementary materials and methods.

### Immunohistochemical staining

Standard immunohistochemistry protocol was performed on paraffin sections used with antibodies listed in the Supplementary Table 1. The methods were described in supplementary materials and methods.

### DNMT inhibition in MDA-MB-231 cells

RG108 is cell-permeable and directly inhibits DNMTs^55^. 5-aza-dC causes DNA demethylation or hemi-demethylation, which can regulate gene expression. Each was solubilized in DMSO at a stock concentration of 10 mM and 1 mg/ml.

### In vitro methylation and identification

Cytosines of PCR fragments were methylated using MSssI and MspI recombinant methyltransferase (NEB, M0226M) according to the recommendations provided by the manufacturer. DNA was purified by gel extraction. Successful methylation was verified by corresponding methylation-sensitive endonuclease restriction.

### Luciferase reporter gene assay

HIF-1α activity was detected by transfecting The 6 × HRE luciferase vector. Methylated and unmethylated PCR products were cloned into the PGL3-enhancer vector. All constructs were confirmed by Sanger sequencing. The *Renilla* luciferase vector was co-transfected and used to normalize transfection efficiency using the dual-luciferase reporter assay kit (E1980, Promega, Madison, WI, USA) according to the manufacturer’s instructions. All data were normalized to *Renilla* luciferase luminescence derived from the co-transfected pRL-SV40 vector (E2231, Promega, Madison, WI, USA).

### Chromatin immunoprecipitation (ChIP) and ChIP-qPCR

To determine whether H3K9ac occupancy on the HIF-1α promoter and first exon leads to HIF-1α gene activity, ChIP was performed as previously described, with minor modifications^56^. Details of data processing are described in supplementary materials and methods.

### Statistical analysis

Statistical analysis was performed using SPSS 16.0 (SPSS Inc., Chicago, IL, USA). A Student’s *t*-test was performed to compare means between experimental and control groups at a single time point. Data were evaluated by the Pearson’s Chi-Square method with SSPS software (Version 16.0) to compare between groups. All data are presented as means ± standard deviations (SD) unless otherwise indicated. Values of *p* < 0.05 were considered statistically significant. Each experiment was performed at least three times.

## Supplementary information

Supplementary Table 1.

Figure S1

Figure S2

Figure S3

Figure S4

Figure S5

Supplementary Figure legends.

## References

[1] Jemal A (2011). Global cancer statistics. CA Cancer J. Clin..

[2] Torre LA (2015). Global cancer statistics, 2012. CA Cancer J. Clin..

[3] Li H (2018). Incidence and mortality of female breast cancer in China, 2014. Zhonghua zhong liu za zhi [Chin. J. Oncol.]..

[4] Irvin WJ, Carey LA (2008). What is triple-negative breast cancer?. Eur. J. Cancer.

[5] Katayama A (2017). Expression patterns of claudins in patients with triple-negative breast cancer are associated with nodal metastasis and worse outcome. Pathol. Int..

[6] Lehmann BD (2011). Identification of human triple-negative breast cancer subtypes and preclinical models for selection of targeted therapies. J. Clin. Invest..

[7] Alluri P, Newman LA (2014). Basal-like and triple-negative breast cancers: searching for positives among many negatives. Surg. Oncol. Clin. N. Am..

[8] Rebecca VW, Amaravadi RK (2016). Emerging strategies to effectively target autophagy in cancer. Oncogene.

[9] Vera-Ramirez, L., Vodnala, S. K., Nini, R., Hunter, K. W., Green, J. E. Autophagy promotes the survival of dormant breast cancer cells and metastatic tumour recurrence. *Nat.* Commun. **9**, 1944 (2018). 10.1038/s41467-018-04070-6 PMC5964069 29789598

[10] Wilson WR, Hay MP (2011). Targeting hypoxia in cancer therapy. Nat. Rev. Cancer.

[11] Harris AL (2002). Hypoxia—a key regulatory factor in tumour growth. Nat. Rev. Cancer.

[12] Semenza GL (2010). Defining the role of hypoxia-inducible factor 1 in cancer biology and therapeutics. Oncogene.

[13] Noman MZ (2015). Hypoxia: a key player in antitumor immune response. A review in the theme: cellular responses to hypoxia. Am. J. Physiol. Cell. Physiol..

[14] Chouaib S, Noman MZ, Kosmatopoulos K, Curran MA (2017). Hypoxic stress: obstacles and opportunities for innovative immunotherapy of cancer. Oncogene.

[15] Kallio PJ, Pongratz I, Gradin K, McGuire J, Poellinger L (1997). Activation of hypoxia-inducible factor 1alpha: posttranscriptional regulation and conformational change by recruitment of the Arnt transcription factor. Proc. Natl. Acad. Sci. USA.

[16] Lin A (2016). The LINK-A lncRNA activates normoxic HIF1alpha signalling in triple-negative breast cancer. Nat. Cell Biol..

[17] Hoffmann C (2018). Hypoxia promotes breast cancer cell invasion through HIF-1alpha-mediated up-regulation of the invadopodial actin bundling protein CSRP2. Sci. Rep..

[18] Luczak MW (2011). Increased expression of HIF-1A and its implication in the hypoxia pathway in primary advanced uterine cervical carcinoma. Oncol. Rep..

[19] Bao L (2018). Methylation of hypoxia-inducible factor (HIF)-1alpha by G9a/GLP inhibits HIF-1 transcriptional activity and cell migration. Nucleic Acids Res..

[20] Koslowski M, Luxemburger U, Tureci O, Sahin U (2011). Tumor-associated CpG demethylation augments hypoxia-induced effects by positive autoregulation of HIF-1alpha. Oncogene.

[21] Gruenbaum Y, Naveh-Many T, Cedar H, Razin A (1981). Sequence specificity of methylation in higher plant DNA. Nature.

[22] Yoder JA, Walsh CP, Bestor TH (1997). Cytosine methylation and the ecology of intragenomic parasites. Trends Genet..

[23] Casadesus J, Low D (2006). Epigenetic gene regulation in the bacterial world. Microbiol. Mol. Biol. Rev..

[24] Pinarbasi E, Elliott J, Hornby DP (1996). Activation of a yeast pseudo DNA methyltransferase by deletion of a single amino acid. J. Mol. Biol..

[25] Ramsahoye BH (2000). Non-CpG methylation is prevalent in embryonic stem cells and may be mediated by DNA methyltransferase 3a. Proc..Natl. Acad. Sci. USA.

[26] Lister R (2009). Human DNA methylomes at base resolution show widespread epigenomic differences. Nature.

[27] Laurent L (2010). Dynamic changes in the human methylome during differentiation. Genome Res..

[28] Lister R (2011). Hotspots of aberrant epigenomic reprogramming in human induced pluripotent stem cells. Nature.

[29] Ziller MJ (2011). Genomic distribution and inter-sample variation of non-CpG methylation across human cell types. PLoS. Genet..

[30] Stadler MB (2011). DNA-binding factors shape the mouse methylome at distal regulatory regions. Nature.

[31] Smith ZD (2012). A unique regulatory phase of DNA methylation in the early mammalian embryo. Nature.

[32] Tomizawa S (2011). Dynamic stage-specific changes in imprinted differentially methylated regions during early mammalian development and prevalence of non-CpG methylation in oocytes. Development.

[33] Ichiyanagi T, Ichiyanagi K, Miyake M, Sasaki H (2013). Accumulation and loss of asymmetric non-CpG methylation during male germ-cell development. Nucleic Acids Res..

[34] Smith ZD, Meissner A (2013). DNA methylation: roles in mammalian development. Nat. Rev. Genet..

[35] Ma H (2014). Abnormalities in human pluripotent cells due to reprogramming mechanisms. Nature.

[36] Feng J, Fan G (2009). The role of DNA methylation in the central nervous system and neuropsychiatric disorders. Int. Rev. Neurobiol..

[37] Okano M, Bell DW, Haber DA, Li E (1999). DNA methyltransferases Dnmt3a and Dnmt3b are essential for de novo methylation and mammalian development. Cell..

[38] Arand J (2012). In vivo control of CpG and non-CpG DNA methylation by DNA methyltransferases. PLoS. Genet..

[39] Lister R (2013). Global epigenomic reconfiguration during mammalian brain development. Science.

[40] Guo JU (2014). Distribution, recognition and regulation of non-CpG methylation in the adult mammalian brain. Nat. Neurosci..

[41] Lachance G (2014). DNMT3a epigenetic program regulates the HIF-2alpha oxygen-sensing pathway and the cellular response to hypoxia. Proc. Natl. Acad. Sci. USA.

[42] Brenet F (2011). DNA methylation of the first exon is tightly linked to transcriptional silencing. PLoS ONE.

[43] Weber M (2007). Distribution, silencing potential and evolutionary impact of promoter DNA methylation in the human genome. Nat. Genet..

[44] Han H (2011). DNA methylation directly silences genes with non-CpG island promoters and establishes a nucleosome occupied promoter. Hum. Mol. Genet..

[45] Nishino K (2011). Non-CpG methylation occurs in the regulatory region of the Sry gene. J. Reprod. Dev..

[46] Barres R (2009). Non-CpG methylation of the PGC-1alpha promoter through DNMT3B controls mitochondrial density. Cell. Metab..

[47] Pilegaard H, Saltin B, Neufer PD (2003). Exercise induces transient transcriptional activation of the PGC-1alpha gene in human skeletal muscle. J. Physiol..

[48] Egan B (2010). Exercise intensity-dependent regulation of peroxisome proliferator-activated receptor coactivator-1 mRNA abundance is associated with differential activation of upstream signalling kinases in human skeletal muscle. J. Physiol..

[49] Pilegaard H, Ordway GA, Saltin B, Neufer PD (2000). Transcriptional regulation of gene expression in human skeletal muscle during recovery from exercise. Am. J. Physiol. Endocrinol. Metab..

[50] Hinshelwood RA (2009). Aberrant de novo methylation of the p16INK4A CpG island is initiated post gene silencing in association with chromatin remodelling and mimics nucleosome positioning. Hum. Mol. Genet..

[51] Li J (2013). Critical role of histone acetylation by p300 in human placental 11beta-HSD2 expression. J. Clin. Endocrinol. Metab..

[52] Bowers EM (2010). Virtual ligand screening of the p300/CBP histone acetyltransferase: identification of a selective small molecule inhibitor. Chem. Biol..

[53] Ivan M (2001). HIFalpha targeted for VHL-mediated destruction by proline hydroxylation: implications for O2 sensing. Science.

[54] Min JH (2002). Structure of an HIF-1alpha—pVHL complex: hydroxyproline recognition in signaling. Science.

[55] Brueckner B (2005). Epigenetic reactivation of tumor suppressor genes by a novel small-molecule inhibitor of human DNA methyltransferases. Cancer Res..

[56] Saleh A, Alvarez-Venegas R, Avramova Z (2008). An efficient chromatin immunoprecipitation (ChIP) protocol for studying histone modifications in Arabidopsis plants. Nat. Protoc..

