# Supplementary material for: Hypomethylation at non-CpG/CpG sites in the promoter of HIF-1α gene combined with enhanced H3K9Ac modification contribute to maintain higher HIF-1α expression in breast cancer
Source: Oncogenesis. 2019 Apr 2;8(4):26. doi: 10.1038/s41389-019-0135-1 (PMC6445832; doi:10.1038/s41389-019-0135-1)
Supplement: Supplementary file 1 — Supplementary Table 1. [file 41389_2019_135_MOESM1_ESM.pdf]

Supplementary Table 1. Antibodies used in this study

Antibody	Cat: #	Company	Con.	Species	
HIF-1a	#14179	CST	1:1000	Rabbit	WB&IHC
DNMT3a	A-1003	Epigentek	1:200	Rabbit	WB
HIF-2a	Ab199	Abcam	1:800	Rabbit	WB&IHC
DNMT3b	Ab16376	Abcam	1:1000	Rabbit	WB&IHC
DNMT1	#5119	CST	1:800	Rabbit	WB
MeCP2	Ab50005	Abcam	1:1000	Mouse	WB
H3K9ac	#9649	CST	1:1000	Rabbit	WB
H3K9ac	#9649	CST	1:50	Rabbit	Chip
DNMT3a	PA-11158	invitrogen	1:200	Rabbit	IHC
GAPDH	TA08	ZSGB-Bio	1:2000	Mouse	WB
β -actin	#3700	CST	1:2000	Mouse	WB
**Secondary antibodies**					
Anti-rabbit IgG,HRP-linked Antidoby	7074S	CST	1:5000	Rabbit	WB
Anti-mouse IgG, HRP-linked Antidoby	7076S	CST	1:5000	Mouse	WB
IgG	#3900	cst	1:5000	Rabbit	Chip
Goat anti Mouse	ZB-2305	ZSGB-Bio	1:3000	Goat	IHC
Goat anti Rabbit	ZB-2301	ZSGB-Bio	1:3000	Goat	IHC
