# Supplementary figures and images for: Hypomethylation at non-CpG/CpG sites in the promoter of HIF-1α gene combined with enhanced H3K9Ac modification contribute to maintain higher HIF-1α expression in breast cancer
Source: Oncogenesis. 2019 Apr 2;8(4):26. doi: 10.1038/s41389-019-0135-1 (PMC6445832; doi:10.1038/s41389-019-0135-1)

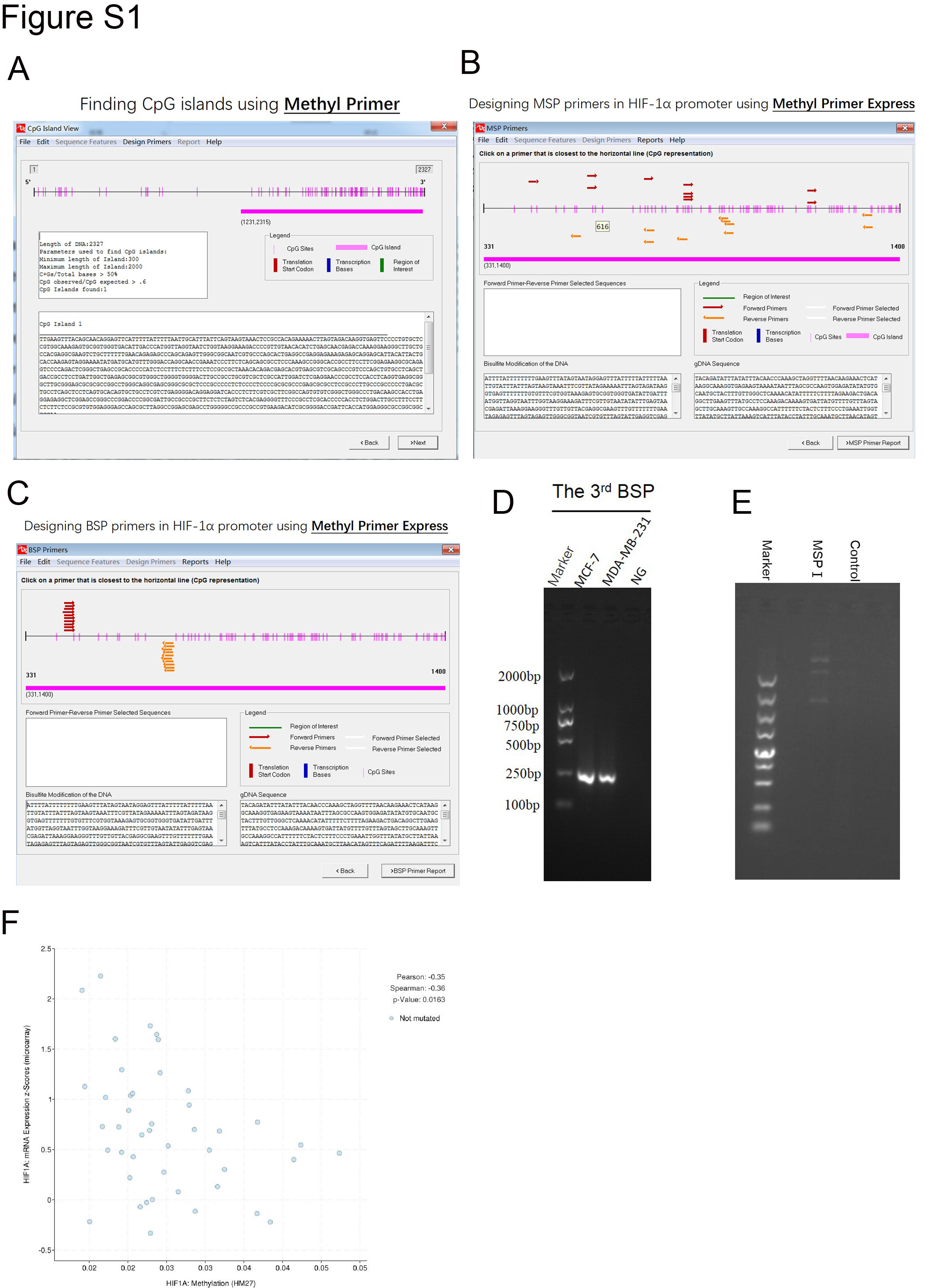

Supplement: Supplementary file 2 — Figure S1 [file 41389_2019_135_MOESM2_ESM.jpg]

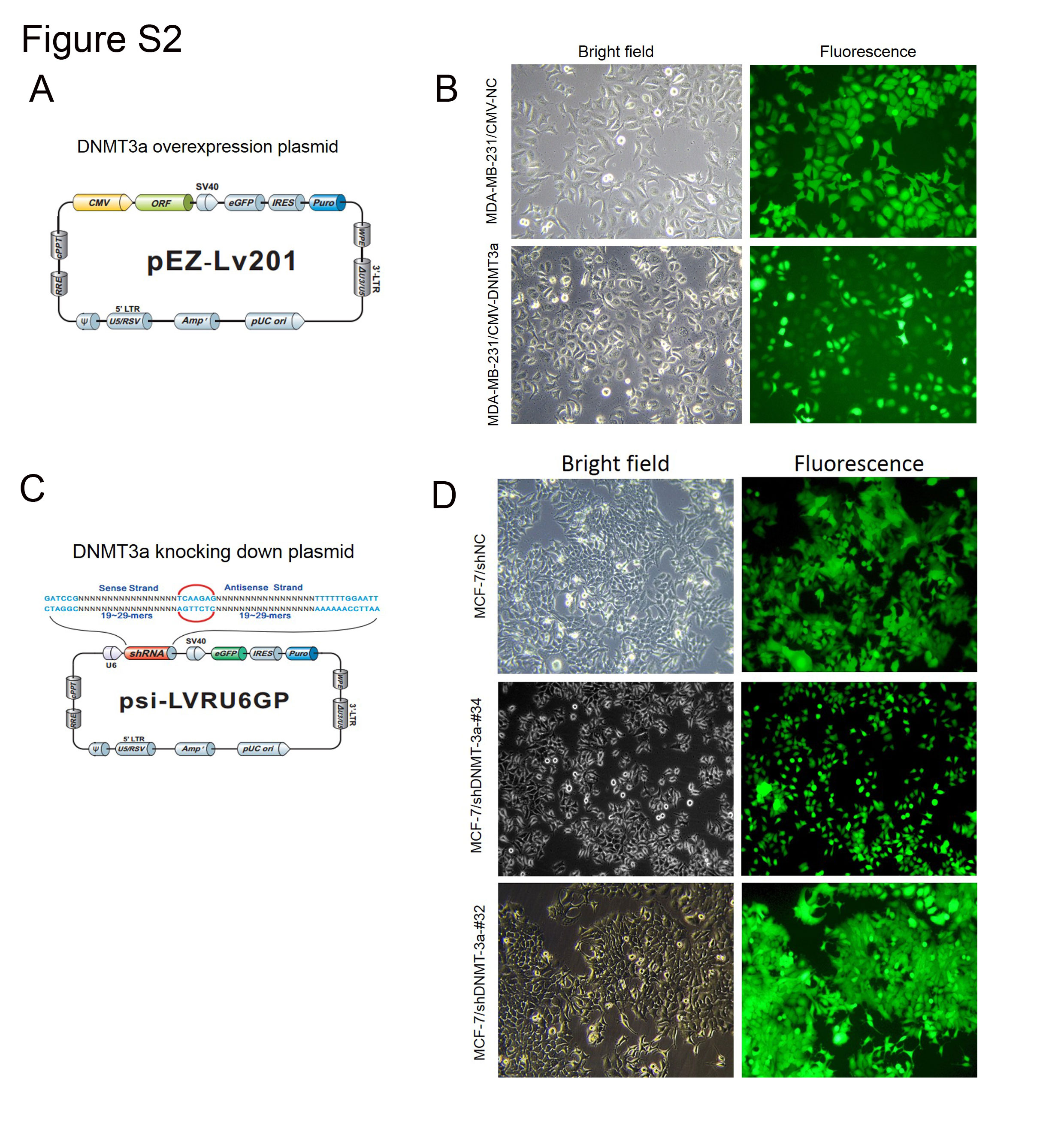

Supplement: Supplementary file 3 — Figure S2 [file 41389_2019_135_MOESM3_ESM.jpg]

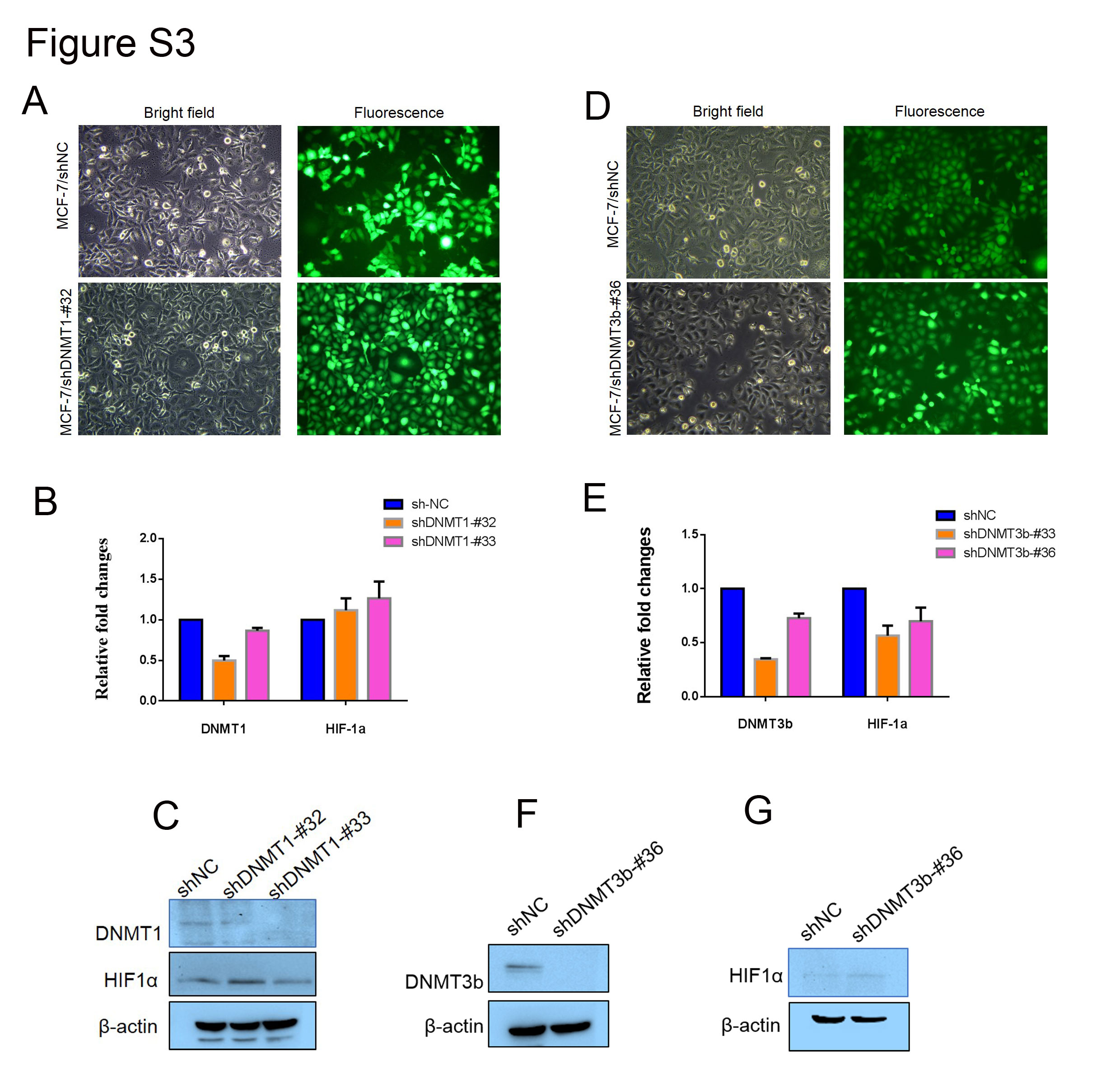

Supplement: Supplementary file 4 — Figure S3 [file 41389_2019_135_MOESM4_ESM.jpg]

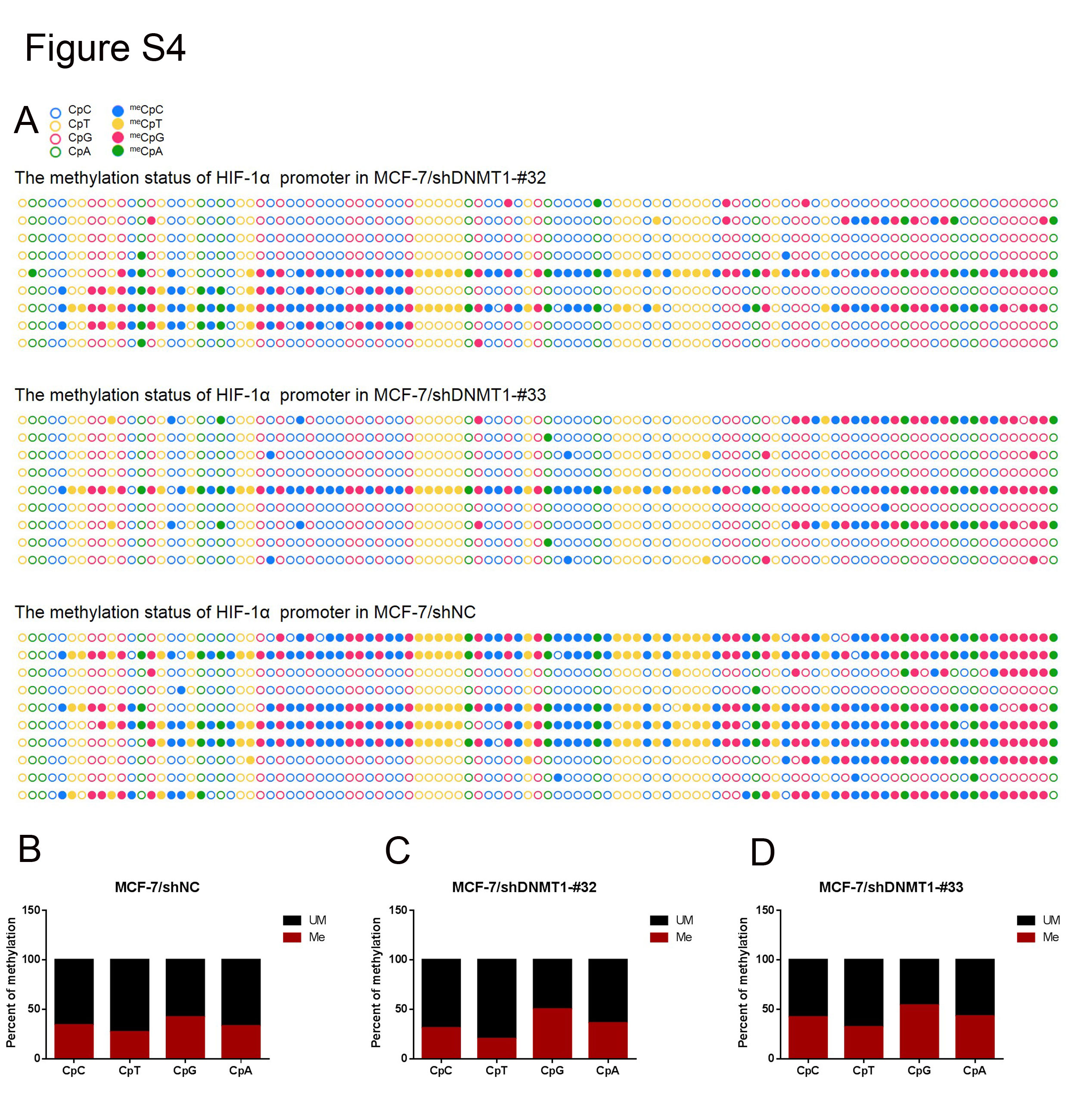

Supplement: Supplementary file 5 — Figure S4 [file 41389_2019_135_MOESM5_ESM.jpg]

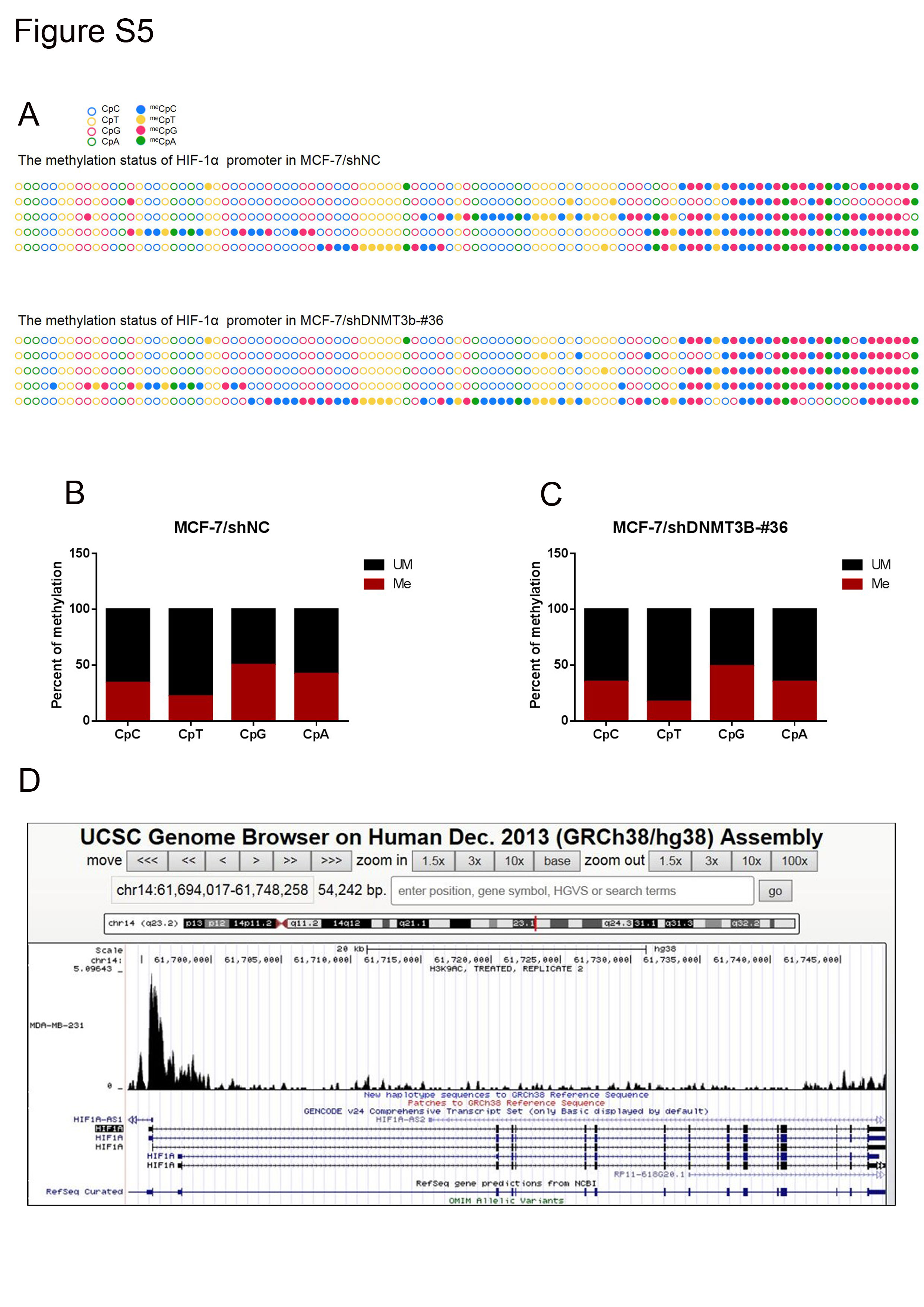

Supplement: Supplementary file 6 — Figure S5 [file 41389_2019_135_MOESM6_ESM.jpg]
