# Supplementary material for: Hypomethylation at non-CpG/CpG sites in the promoter of HIF-1α gene combined with enhanced H3K9Ac modification contribute to maintain higher HIF-1α expression in breast cancer
Source: Oncogenesis. 2019 Apr 2;8(4):26. doi: 10.1038/s41389-019-0135-1 (PMC6445832; doi:10.1038/s41389-019-0135-1)
Supplement: Supplementary file 7 — Supplementary Figure legends. [file 41389_2019_135_MOESM7_ESM.pdf]

**Supplementary materials**

**Supplementary Figure legends**

**Figure S1: Methyl Primer Express Software v1.0 is used to analyze CpG island of HIF-1α gene promoter and designed MSP and BSP primers.**

(A): Methyl primer Express Software V1.0 is used to analyze and predict CpG islands in the HIF-1α promoter. (B): The primers for Methylation Specific PCR (MSP) are designed to target the methylation status at specific CpG sites using Methyl Primer Express Software v1.0. (C): The primers for bisulfite sequencing PCR (BSP) are designed to target the methylation status at specific CpG sites using Methyl Primer Express Software v1.0. (D): BSP is performed using the DNA as the templet, which extracted from MCF-7 and MDA-MB-231 cells and treated with sodium bisulfite. (E): The successful in vitro methylation was verified by corresponding methylation sensitive restriction endonucleases. (F): cBioportal data (TCGA, Cells (2015)) show significant negative correlations between HIF-1α promoter methylation and mRNA expression level in 107 basal-like breast cancer samples (PAM50).

**Figure S2: Constructing and making DNMT3a overexpression or knockdown lentivirus.**

(A): The DNMT3a overexpressing lentivirus vector map. (B): The MDA-MB-231 cells are successfully infected with DNMT3a overexpressing lentivirus, which is shown by GFP expression. (C): The DNMT3a knockdown lentivirus vector map. (D): The MCF-7 cells are successfully infected with DNMT3a knockdown lentivirus, which is shown by GFP expression.

**Figure S3: Constructing and making DNMT1 and DNMT3b knockdown lentivirus, as well as confirming the efficiency of knockdown.**

(A): The DNMT1 knockdown lentivirus constructs are engineered, and

successful transfection is shown by GFP expression. (B): RT-PCR confirms that DNMT1 mRNA expression is downregulated in MCF-7 cells transfected with two knockdown plasmids: shDNMT1-#32 and #33. (C): Western blotting confirms that DNMT1 protein expression is downregulated in MCF-7 cells transfected with shDNMT1-#32 and #33. (D): The DNMT3b knockdown lentivirus constructs are engineered, and successful transfection is shown by GFP expression. (E): RT-PCR confirms that DNMT3b mRNA expression is downregulated in MCF-7 cells transfected with knockdown plasmid, shDNMT1-#36, whereas no significant change is observed in expression level of HIF-1α mRNA. (F): Western blotting confirms that DNMT3b protein expression is downregulated in MCF-7 cells transfected with shDNMT3b-#36, whereas no significant change is observed in expression level of HIF-1α protein. Data are presented as the means ± SD of three independent experiments. * $p < .05$, ** $p < .01$ and *** $p < .001$ (Student's *t*-test) as compared to control cells.

**Figure S4: DNMT1 does not involve in the methylation at CpC and non-CpC loci within promoter and first exon of HIF-1α gene in breast cancer cells.**

(A): Comparison of bisulfite sequencing results after knocking down DNMT1 in MCF-7 cells (containing two target sites). Filled circles represent methylation, and blank circles represent nonmethylation. (B&C): Calculating and comparing the methylation frequencies at CpC, CpT, CpG and CpA loci within the promoter and first exon of HIF-1α gene after knocking down DNMT1. Data are presented as the means ± SD of three independent experiments. * $p < .05$, ** $p < .01$ and *** $p < .001$ (Student's *t*-test) as compared to control cells.

**Figure S5: DNMT3b does not involve in the methylation at CpC and non-CpC loci within promoter and first exon of HIF-1α gene in breast cancer cells.**

(A): Comparison of bisulfite sequencing results after knocking down DNMT3b

in MCF-7 cells. Filled circles represent methylation, and blank circles represent nonmethylation. (B&C): Calculating and comparing the methylation frequencies at CpC, CpT, CpG and CpA loci within the promoter and first exon of HIF-1α gene after knocking down DNMT3b. (D): UCSC browser shows H3K9ac binding to the HIF-1α promoter region around TSS. Data are presented as the means ± SD of three independent experiments. * $p < .05$, ** $p < .01$ and *** $p < .001$ (Student's *t*-test) as compared to control cells.
